# Antagonistic Pleiotropy in the Bifunctional Surface Protein FadL (OmpP1) during Adaptation of Haemophilus influenzae to Chronic Lung Infection Associated with Chronic Obstructive Pulmonary Disease

**DOI:** 10.1128/mBio.01176-18

**Published:** 2018-09-25

**Authors:** Javier Moleres, Ariadna Fernández-Calvet, Rachel L. Ehrlich, Sara Martí, Lucía Pérez-Regidor, Begoña Euba, Irene Rodríguez-Arce, Sergey Balashov, Ester Cuevas, Josefina Liñares, Carmen Ardanuy, Sonsoles Martín-Santamaría, Garth D. Ehrlich, Joshua Chang Mell, Junkal Garmendia

**Affiliations:** a Instituto de Agrobiotecnología, CSIC-Universidad Pública Navarra-Gobierno Navarra, Mutilva, Spain; b Department of Microbiology and Immunology, Drexel University College of Medicine, Philadelphia, Pennsylvania, USA; c Center for Genomic Sciences, Drexel University College of Medicine, Philadelphia, Pennsylvania, USA; d Center for Advanced Microbial Processing, Drexel University College of Medicine, Philadelphia, Pennsylvania, USA; e Center for Surgical Infections and Biofilms, Institute for Molecular Medicine and Infectious Disease, Drexel University College of Medicine, Philadelphia, Pennsylvania, USA; f Centro de Investigación Biomédica en Red de Enfermedades Respiratorias (CIBERES), Madrid, Spain; g Microbiology Department, Hospital Universitari Bellvitge, University of Barcelona, IDIBELL, Barcelona, Spain; h Department of Structural and Chemical Biology, Centro de Investigaciones Biológicas, CIB-CSIC, Madrid, Spain; i Pneumology Department, Hospital Universitari Bellvitge, University of Barcelona, IDIBELL, Barcelona, Spain; j Department of Otolaryngology, Head and Neck Surgery, Drexel University College of Medicine, Philadelphia, Pennsylvania, USA; The Sanger Institute

**Keywords:** CEACAM1, FadL/OmpP1, *Haemophilus influenzae*, adaptive evolution, antagonistic pleiotropic, chronic obstructive pulmonary disease, comparative genomics, convergent evolution, free fatty acids, *in situ* evolution

## Abstract

Tracking bacterial evolution during chronic infection provides insights into how host selection pressures shape bacterial genomes. The human-restricted opportunistic pathogen nontypeable Haemophilus influenzae (NTHi) infects the lower airways of patients suffering chronic obstructive pulmonary disease (COPD) and contributes to disease progression. To identify bacterial genetic variation associated with bacterial adaptation to the COPD lung, we sequenced the genomes of 92 isolates collected from the sputum of 13 COPD patients over 1 to 9 years. Individuals were colonized by distinct clonal types (CTs) over time, but the same CT was often reisolated at a later time or found in different patients. Although genomes from the same CT were nearly identical, intra-CT variation due to mutation and recombination occurred. Recurrent mutations in several genes were likely involved in COPD lung adaptation. Notably, nearly a third of CTs were polymorphic for null alleles of *ompP1* (also called *fadL*), which encodes a bifunctional membrane protein that both binds the human carcinoembryonic antigen-related cell adhesion molecule 1 (hCEACAM1) receptor and imports long-chain fatty acids (LCFAs). Our computational studies provide plausible three-dimensional models for FadL’s interaction with hCEACAM1 and LCFA binding. We show that recurrent *fadL* mutations are likely a case of antagonistic pleiotropy, since loss of FadL reduces NTHi’s ability to infect epithelia but also increases its resistance to bactericidal LCFAs enriched within the COPD lung. Supporting this interpretation, truncated *fadL* alleles are common in publicly available NTHi genomes isolated from the lower airway tract but rare in others. These results shed light on molecular mechanisms of bacterial pathoadaptation and guide future research toward developing novel COPD therapeutics.

## Abstract (precis)

Nontypeable Haemophilus influenzae is an important pathogen in patients with chronic obstructive pulmonary disease (COPD). To elucidate the bacterial pathways undergoing *in vivo* evolutionary adaptation, we compared bacterial genomes collected over time from 13 COPD patients and identified recurrent genetic changes arising in independent bacterial lineages colonizing different patients. Besides finding changes in phase-variable genes, we found recurrent loss-of-function mutations in the *ompP1* (*fadL*) gene. We show that loss of OmpP1/FadL function reduces this bacterium’s ability to infect cells via the hCEACAM1 epithelial receptor but also increases its resistance to bactericidal fatty acids enriched within the COPD lung, suggesting a case of antagonistic pleiotropy that restricts Δ*fadL* strains’ niche. These results show how H. influenzae adapts to host-generated inflammatory mediators in the COPD airways.

## INTRODUCTION

Bacterial pathogens evolve during long-term chronic infections (1), and identifying the genetic changes that arise in bacteria over time within hosts is a powerful means of deciphering host selective pressures acting on bacteria that colonize new niches and the specific adaptations that allow bacteria to persist (2–4). Dissecting the genetics of bacterial adaptation is also key to understanding and predicting the emergence of antimicrobial resistance and immune evasion traits within bacterial populations, as well as for identifying new interventional targets.

Colonization of the human lower airways by opportunistic pathogens of environmental origin leads to the irreversible progression of major respiratory diseases, such as cystic fibrosis (CF), and bacterial evolution during long-term lung infection has been extensively analyzed in this context (2, 4, 5). In particular, genomic analyses of serially collected isolates of Pseudomonas aeruginosa and Burkholderia species have provided *in vivo* evidence of adaptation during long-term infection of CF lungs (3, 4, 6–13). In contrast, we only scarcely understand how different members of the human microbiome adapt to new niches within the human body and become pathogens, and our knowledge is limited largely to Staphylococcus aureus evolutionary dynamics during CF lung infection and transitions from carriage to invasive disease (14–18).

Chronic obstructive pulmonary disease (COPD), the fourth leading cause of death globally, whose primary risk factor is smoking, is an irreversible airflow obstruction accompanied by emphysema, fibrosis, neutrophil airway infiltration, mucus hypersecretion, inflammation, and long-term lower airway colonization by opportunistic pathogens (19). Though nontypeable Haemophilus influenzae (NTHi) is typically a benign commensal of the human upper airways, it is also an opportunistic pathogen commonly isolated from the lower airways of COPD patients during both exacerbations and clinically stable periods, and it is responsible for 20 to 30% of all acute COPD exacerbations. NTHi persists within COPD patient lungs for months to years, contributing to chronic airway inflammation that results in worsening of symptoms and speeds disease progression (19).

NTHi isolates are extremely diverse both phenotypically (20) and genomically (21–23). Moreover, individuals are often colonized by multiple distinct strains (24), distinguished by hundreds of gene possession differences and 10s of thousands of single-nucleotide variants (SNVs) (22, 25–31). In addition, NTHi is naturally competent (able to take up and recombine DNA from relatives into their chromosome) and possesses numerous phase-variable genes (28, 32–35), traits that likely contribute to NTHi’s ability to adapt and persist within COPD patient lungs. Indeed, the recent whole-genome sequencing of a unique set of prospectively collected NTHi strains from COPD patients revealed frequent genetic variation due to slipped-strand mispairing in simple sequence repeats (SSRs) and diversifying selection in several candidate vaccine antigens during NTHi persistence in the human airways (36).

In this study, we addressed molecular genetic changes underlying bacterial adaptation to the COPD airway by investigating the genetic variants arising from within-patient evolution of NTHi in an independent cohort of patients. We identify recurrent polymorphisms in several genes and provide experimental evidence for the biological significance of such convergent variation in the *ompP1* (also called *fadL*) gene. Overall, our results provide novel insights into the evolution of a human-restricted respiratory commensal to be a persistent pathogen of the lower airway.

## RESULTS

### Longitudinal collection of NTHi genomes from COPD patients.

**FIG 1:**
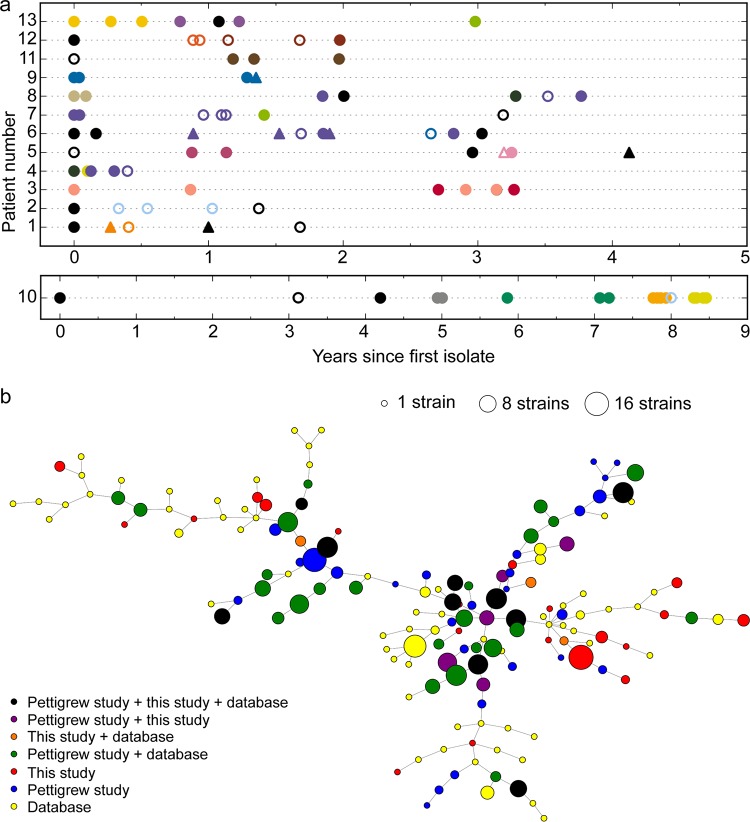
(a) Overview of longitudinal sampling of NTHi isolates from COPD patients; the strains consisted of 92 isolates collected over 1 to 9 years from 13 COPD patients (4 to 18 isolates per patient; strains were collected between the years 2000 and 2014) (Table 1; see also Data Set S1a and b in the supplemental material). Axes indicate patient identifier (ID) numbers and the time of sampling for each isolate. Symbols indicate whether the isolate was collected outside an acute exacerbation (triangles) or not (circles). Colors indicate clonal type (CT) based on 309 single-copy core protein-coding genes. Black is reserved for CTs collected only once. Filled symbols indicate full-length *fadL*; open symbols indicate a truncated *fadL* allele. (b) Minimum-spanning tree (MST) of CTs across all genomes in the study. Nodes represent individual CTs (collapsed from the genotype-based nodes seen in Fig. S3), and edges indicate links between closely related CTs. Node size indicates the number of strains across the data set belonging to that CT, and node color indicates to which data set strains in each CT belong. Primary colors indicate that CTs contain a strain only from this work (red), from the work of Pettigrew et al. (36) (blue), or from other works (yellow). Mixtures of the primary colors indicate >1 strain from two data sets, with black indicating CTs containing strains from all three sets of strains.

To identify natural genetic variation in bacterial genomes associated with adaptation to the COPD lung, we serially collected NTHi isolates from a set of COPD patients over time and sequenced and comparatively analyzed their genomes. Sputum samples were collected from COPD patients during exacerbations requiring hospitalization or at primary health care routine visits; NTHi colonies were phenotypically identified and isolated. Thirteen patient series were selected on the basis of the presence of four or more longitudinally sampled NTHi isolates, as well as the presence of more than one independent isolate of the same pulsed-field gel electrophoresis (PFGE) type, suggesting persistent long-term infection by that strain. Sampled subjects were Spanish male smokers or ex-smokers; clinical information on each subject is summarized in Table 1. The bacterial strain collection consists of 92 isolates, with 4 to 18 isolates per patient, collected over a period ranging from 1 to 9 years (Fig. 1a). Details on each isolate are summarized in Data Set S1a and b in the supplemental material.

**TABLE 1:** Information on subjects included in this study^*a*^

Patient	Yr of birth	Age at first NTHi isolation (yr)	No. of AE of COPD/yr	COPD status	% FEV_1_ (mo and/or yr^*b*^)	No. of packs/yr
1	1951	61	5	Gold IV	26.1 (July 2012)	40 (ex-smoker)
2	1935	77	3	Gold III	41 (August 2012)	ND (ex-smoker)
3	1912	88	ND	ND	ND	ND (ex-smoker)
4	1945	67	ND	Gold II	53 (November 2012)	50 (ex-smoker)
5	1933	77	5	Gold II	52 (October 2012)	40 (ex-smoker)
6	1947	64	7	Gold IV	26 (October 2013)	30 (ex-smoker)
7	1963	47	3	Gold II	55 (November 2011)	>20 (smoker)
8	1939	70	3	Gold II	65 (May 2009)	ND (ex-smoker)
9	1921	90	2	Gold I	91 (2007)	ND (ex-smoker)
10	1946	59	ND	Gold III	40 (March 2008)	ND (ex-smoker)
11	1946	66	>3	Gold IV	29 (November 2012)	100 (ex-smoker)
12	1937	73	3	Gold III	35 (March 2011)	67 (ex-smoker)
13	1926	84	7	Gold III	47 (2007)	20 (ex-smoker)

a AE, acute exacerbations; FEV_1_, forced expiratory volume in the 1st second; ND, not determined.

b Date of spirometry test used to assign COPD Gold stage.

10.1128/mBio.01176-18.8 DATA SET S1 (a) Information on patient visits when the NTHi COPD isolates were collected. (b) NTHi isolates collected for this study, organized by clonal type (CT), as determined by goeBurst using allelic identities at 309 core genes. Assemblies for NTHi strains shown in bold were used as reference sequences for short-read alignments and intra-CT variant calling. If a PacBio reference was unavailable for a given CT, the Illumina assembly with the highest *N*_50_ was used as the reference. Biosample ID, PFGE profile, *in silico* MLST determination, and *fadL* allele number are included. (c) Sequencing, assembly, and annotation metrics for the 92 new NTHi genomes collected from COPD patients for this study (format, .xlsx). (d) Results of the Pacific Biosciences RS_modification_motif_analysis pipeline for 10 isolates sequenced by PacBio. (e) FadL allelic variants encoding full-length (upper) and truncated (lower) proteins. Download Data Set S1, XLSX file, 0.1 MB. Copyright © 2018 Moleres et al. 2018 Moleres et al. This content is distributed under the terms of the Creative Commons Attribution 4.0 International license.

The genomes of all isolates were subjected to Illumina-based shotgun whole-genome sequencing, and a subset of 17 isolates were also sequenced using the long-read Pacific Biosciences RSII technology (PacBio) to serve as complete reference sequences. Genome assembly, gene annotation, taxonomic classification, and *in silico* multilocus sequencing typing (MLST) were performed. Summary metrics are shown in Data Set S1c; results of DNA modification motif analysis for 10 PacBio assemblies are shown in Data Set S1d. Assemblies from the two sequencing methods showed high overall agreement (Fig. S1; see also Materials and Methods).

10.1128/mBio.01176-18.2 FIG S1 PacBio versus Illumina. Each of the 17 PacBio genomes were also sequenced on an Illumina sequencer, so the 17 pairs of assemblies were compared. (a) Each subplot compares the feature counts for the annotated genomes. Strains are on the *x* axis, feature counts are on the *y* axis, Illumina genomes are indicated by green dots, and PacBio genomes are indicated by blue dots. Notably, P602, although a single-contig assembly, failed to circularize with Circlator, resulting in a terminal duplication of the 23S gene. (b) Roary computed the pangenome for the 17 pairs of assemblies as described in Materials and Methods. In each subgraph, the *x* axis is the number of strain pairs and the *y* axis is the number of gene clusters from Roary. From left to right, (i) “only_present_in_illumina” means genes that were present in the Illumina assembly but not in the PacBio assembly (most genes were present in both assemblies as indicated by the bar for zero strain pairs); (ii) “only_present_in_pacbio” means genes that were present in the PacBio assembly but not in the Illumina assembly; (iii) “pacbio_has_more_copies” means, in many cases, that multiple genes from a genome belong to the same gene cluster (this subplot counts the number of strains where the PacBio assembly had a higher copy number for the gene cluster than did the Illumina assembly); (iv) the “illumina_has_more_copies” subplot counts the number of strains where the Illumina assembly had a higher copy number for the gene cluster than the PacBio assembly; (v) “absent_in_both” means genes that were absent in both assemblies of some strains (these are more likely to be true absences instead of assembly artifacts); and (vi) “present_and_equal_copy_number_in_both” means genes with the same copy number in both assemblies. Download FIG S1, PDF file, 0.9 MB. Copyright © 2018 Moleres et al. 2018 Moleres et al. This content is distributed under the terms of the Creative Commons Attribution 4.0 International license.

### Gene clustering and phylogenomic analysis.

To provide context to these new COPD genomes for comparative genomic analyses, we combined our genome collection with 391 publicly available H. influenzae assemblies (of which 210 were from a recent large study of NTHi in COPD patients by Pettigrew et al. [36], 28 of which were of strains collected from COPD patients or patients with other lower airway infections, and the remaining 153 were non-COPD patient isolates), along with 35 representative genomes from sister species H. haemolyticus and *H. parainfluenzae*. Pan-genome analysis was conducted by clustering together homologous protein-coding genes using the pan-genome analysis pipeline Roary (3), resulting in 6,453 total homologous gene clusters among H. influenzae assemblies, of which 1,305 were present in ≥95% of assemblies and 4,505 were present only in <15% of assemblies (Fig. S2; Data Set S2).

**FIG 2:**
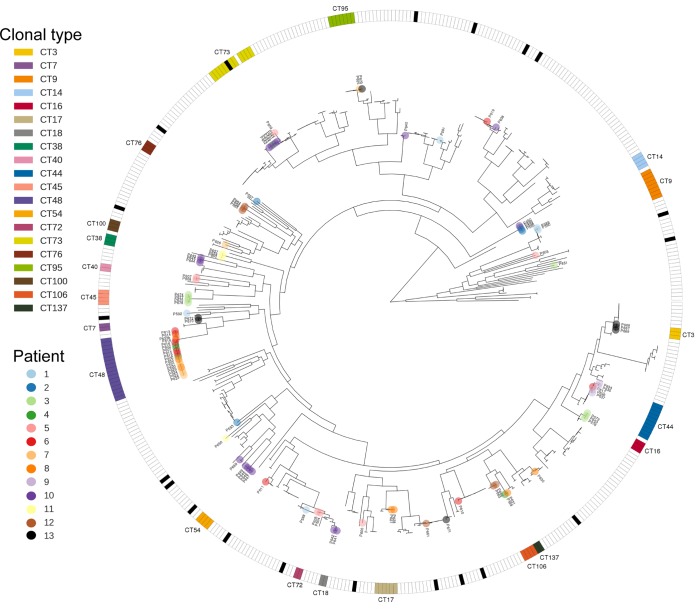
Species-level phylogenetic tree, based on a concatenation of 309 core gene alignments. This tree contains 483 H. influenzae genomes, including 210 of the genomes from the study of Pettigrew et al. (36), 181 publicly available genomes, and 92 genomes from this study. The patient ID is indicated by node color, whereas CT is indicated by the color in the outer ring, as in Fig. 1a. Only COPD isolates sequenced for this study are labeled; Fig. S4a and b, respectively, show an unrooted phylogeny, including H. haemolyticus and H. parainfluenzae outgroups, and a fully labeled rectangular tree that includes bootstrap results.

10.1128/mBio.01176-18.3 FIG S2 Heatmap of gene content of H. influenzae strains. Thirty-five outgroup genomes were left out. Each row in the heatmap is a strain. Each column is a gene from the pangenome. Dark blue indicates that the gene was annotated in the strain, and light yellow indicates that the gene was not annotated in the strain. Strains (rows) are ordered based on the phylogram on the left, and genes (columns) are ordered based on hierarchical clustering. The phylogram on the left is as described for Fig. 2 and Fig. S4b. Download FIG S2, PDF file, 1.4 MB. Copyright © 2018 Moleres et al. 2018 Moleres et al. This content is distributed under the terms of the Creative Commons Attribution 4.0 International license.

10.1128/mBio.01176-18.9 DATA SET S2 Results of Roary-based gene possession analysis. Split paralogous clusters were collapsed into homologous clusters, as per Materials and Methods. Following Roary format, distinct collapsed group IDs and gene annotations are tab delimited within comma-separated quoted strings. A single additional column was added denoting “cluster ID number” for convenient cross-referencing. (format, .csv). This includes only the gene possession results for our strains; a full table is available upon request. Download Data Set S2, CSV file, 2.9 MB. Copyright © 2018 Moleres et al. 2018 Moleres et al. This content is distributed under the terms of the Creative Commons Attribution 4.0 International license.

For molecular typing and clustering of closely related isolates into clonal types (CTs), besides performing PFGE and MLST analyses, we applied the goeBurst algorithm in PHYLOViZ to allele assignments that we made from 309 core protein-coding genes shared by all genomes, including the sister species. For each single-copy core gene, unique nucleotide sequences were defined as distinct alleles, and strings of allele identities (IDs) for each isolate were used as an MLST-like input. Strains with fewer than 15 allelic differences across all core genes were clustered together, and each resulting connected component was assigned an arbitrary CT number (Materials and Methods; Fig. S3). So, CTs are defined as closely related groups of strains but allow some recombination. A clear-cut correlation between CTs and MLSTs was observed, since strains grouped by CT the same way as they grouped by MLST (Data Set S1b), but the former offers higher resolution for distinguishing relationships among CTs.

10.1128/mBio.01176-18.4 FIG S3 Minimum-spanning tree (MST) of database strains and strains sequenced for this study generated by goeBurst (threshold =15 allelic differences in core genes). Size indicates the number of strains with identical genotypes, and color indicates assignment to a particular CT. Only CTs containing 1 or more strain from the 92 NTHi genomes reported here are given a color. Black nodes are CTs that appear only once. Edge lengths all have a fixed length. Download FIG S3, PDF file, 1.5 MB. Copyright © 2018 Moleres et al. 2018 Moleres et al. This content is distributed under the terms of the Creative Commons Attribution 4.0 International license.

Overall, 205 CTs were detected. H. influenzae accounted for 175 of the CTs, including 40 of which appeared in our COPD collection. Twenty of the COPD CTs contained more than one isolate from within our collection, and so the 72 COPD isolates belonging to these 20 CTs formed the basis for the search for recurrent genomic changes (described below). Figure 1b shows a minimum-spanning tree (MST) of all CTs, indicating whether strains belonging to that CT were from this study, the Pettigrew et al. study (36), another study, or some combination. These results indicate that many CTs can be found in both Spanish and American COPD patients, as well as in altogether distinct subjects. Nearly half of the H. influenzae CTs across the data set had only a single isolate (*n* = 87), suggesting that many CTs remain unobserved.

Consistently with results from clonal typing, the species-level phylogenetic tree (built from alignments of all protein-coding homologs with at most one copy per strain) illustrated several findings (Fig. 2; unrooted version with outgroups in Fig. S4a; fully labeled rectangular version in Fig. S4b). (i) Individual patients were colonized by numerous genetically diverse strains over time, though diversity at any given time point remains unclear (24, 32, 37–40). High polyclonality and a high average degree of divergence among CTs contrasts with studies of environmentally acquired opportunistic pathogens, which have typically supported colonization by single CTs followed by subsequent diversification (3, 6, 41). (ii) Groups of closely related isolates were collected both within and among patient series, and they sometimes included isolates from public databases with diverse clinical and geographical origins. (iii) Several instances of probable interpatient transmission were observed (CT14, -44, -48, -73, -95, and -137).

10.1128/mBio.01176-18.5 FIG S4 Species-level phylogeny of NTHi. (a) To identify the root of the H. influenzae phylogeny, an unrooted phylogeny of H. influenzae, H. haemolyticus, and *H. parainfluenzae* strains was produced. Twelve H. haemolyticus and 18 *H. parainfluenzae* genomes from NCBI were annotated with Prokka and combined with 186 database strains (excluding those from the Pettigrew et al. study [36]) and the 92 strains sequenced for this study. Genes were clustered using Roary, codon-aware alignments of genes with at most one copy per strain were concatenated, and a phylogeny was created using RAxML. The 92 strains sequenced for this study are indicated by red dots and are distributed among the database H. influenzae strains. (b) Phylogram of the 483 H. influenzae genomes, including 210 of the genomes from the work of Pettigrew et al. (36). The phylogram was rooted based on the location of the H. haemolyticus and *H. parainfluenzae* strains in panel a; these and five NCBI sequences labeled as H. influenzae but clearly clustering with H. haemolyticus were used to root but were not drawn in the phylogeny. Terminal nodes corresponding to strains from this study are colored by patient ID number. Interior nodes are labeled with their bootstrap values if the values are <90. The three color bars to the right of the phylogram describe properties of the strains. (a) broken_fadL strains have a red bar. (b) Data set strains were either part of this study, the Pettigrew et al. study (36), another lower respiratory isolate, or another non-lower respiratory isolate. (c) “clonal_type,” each CT containing one or more strains sequenced for this study was assigned a color. All strains (this study and database strains) belonging to these CTs were colored, and the CTs were labeled to the right of this color bar. Download FIG S4, GIF file, 1.1 MB. Copyright © 2018 Moleres et al. 2018 Moleres et al. This content is distributed under the terms of the Creative Commons Attribution 4.0 International license.

**FIG 3:**
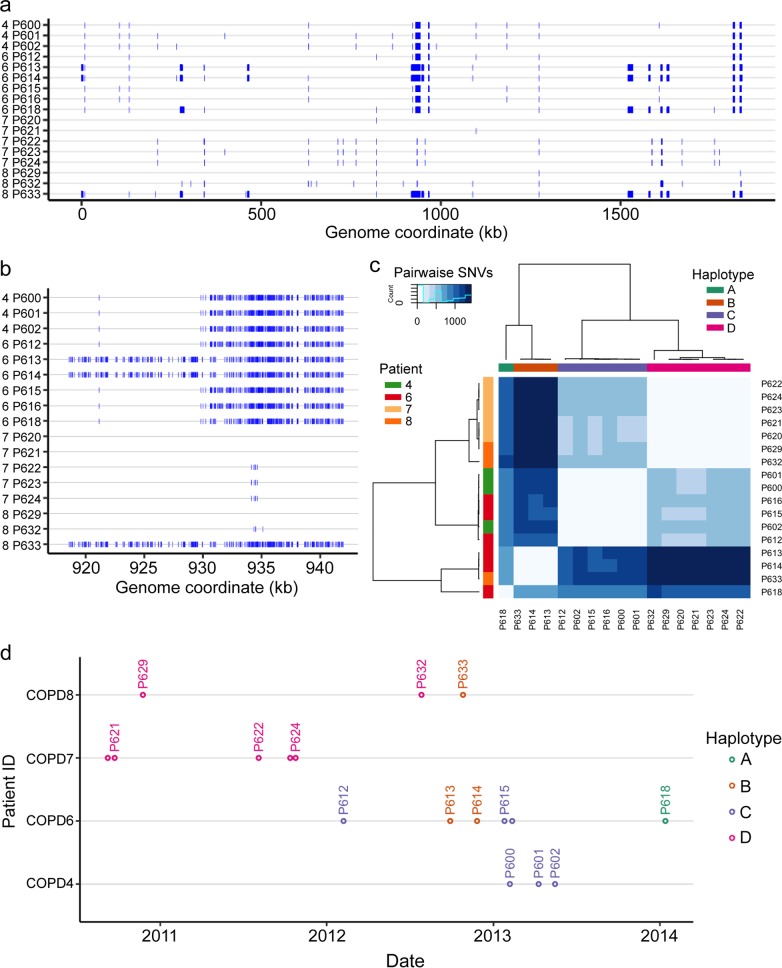
Genetic variation within clonal type 48 (CT48). (a) Whole-genome view of all short variants detected for the 17 isolates in CT48, collected from 4 patients, against the PacBio reference assembly for isolate P621 (the *x* axis shows the P621 genome coordinate; the left-hand side of the *y* axis shows results for patients 4, 6, 7, and 8; blue hatch marks indicate SNVs and short indels). Blocks are due to numerous closely spaced variants, which resemble natural transformation events seen in the laboratory. (b) Zoomed-in view at 920 to 940 kb, showing that recombination tracts in this region likely had independent donor sources. (c) Heatmap of pairwise variant counts among CT48 isolates, showing 4 distinct haplotypes, mostly due to recombination tracts distinguishing the isolates. P618 in haplotype A is more similar to haplotypes B and C than they are to each other and, as seen above, appears to be a hybrid of the two. (d) Time course of all isolates belonging to CT48, encompassing strains collected from four patients.

Most notably, for CT48, 17 isolates were found across four patients, all of whom live within a 400-m radius, effectively neighbors. Furthermore, patients 4 and 6 were hospitalized on the same floor of the hospital in April 2011, patients 4 and 7 were hospitalized on the same floor in December 2012 and November 2013, and patients 6 and 7 were in the emergency unit at the same time in December 2012. No such relationship was found for patients 7 and 8 or 6 and 8. These observations, along with SNV differences among this subset of strains, suggest likely transmission links among these patients, as shown in the time course in Fig. 3 (Data Set S1a and b), though we cannot exclude the possibility that there may be indirect links through medical professionals, mutual friends, or relatives. Alternatively, since the relative abundance of CTs circulating in the population remains unknown, CT48 may simply be common in the local population.

### Recurrent genetic changes among clonal types, especially in genes encoding membrane-associated functions.

Polymorphisms among isolates of the same CT, especially when collected from the same patient, point to recent mutations or recombination events that may contribute to the bacterium’s adapting to the COPD lung environment. Because a single isolate was collected at each sampling time point from what may be polyclonal populations, we did not treat earlier isolates as having parental genotypes and later ones as having derived genotypes (41, 42). Nevertheless, independent polymorphisms at the same locus in different CTs and different patients might be indicative of adaptive evolution (or, alternatively, hotspots of mutation or recombination).

To rank genes by evidence of recurrent changes in distinct CTs, we first identified genetic variants (SNVs, short indels, and gene possession differences) that occurred within each of the 20 multi-isolate CTs. This showed that intraclonal genetic variation was characterized by relatively few genetic differences affecting a small number of loci, either as isolated point mutations or clusters of closely spaced genetic variants, characteristic of natural-transformation events (1, 34, 43, 44) (Fig. 3; Data Set S3). We next classified variants based on their impact on protein coding and ranked homologous protein-coding gene clusters by the number of CTs affected by intra-CT polymorphisms. Of 6,453 total gene clusters, 299 were polymorphic for short variants in one or more of the 20 multi-isolate CTs. Of these, only 15 gene clusters had intra-CT polymorphisms in three or more CTs (Table 2) and only 5 clusters had high-impact (frameshift, nonsense, or nonconservative missense) short variants in three or more CTs. The top hits in Table 2 affected by high-impact variants encoded membrane proteins, enzymes determining lipooligosaccharide (LOS) structure, and a DNA methyltransferase subunit. Several of these are known to be phase variable due to SSRs, whose number determines if the reading frame is open (*lic2A*, *oafA*, *losA*, *hsdM3*) (45–47). The frequent frameshift variants arising within CTs in these genes are due to copy number changes in these repeats due to slip-strand mispairing during replication (Data Set S3). This observation nicely corroborates the recent one by Pettigrew et al. with regard to an independent set of COPD isolates (36). Several hits in Table 2 were also found in the Pettigrew et al. COPD study (36), including DNA methylase, LOS-related glycosyltransferase, acetyltransferase, and a TonB-dependent receptor. Also among top hits were several genes affected by dozens of low-impact intra-CT variants (conservative missense and synonymous variants) affecting the same contiguous gene cluster (e.g., *hgpB*, *hgpC*, and *tbp2*), which were due to overlapping recombination tracts in different CTs (Fig. 3) and potentially indicate loci under diversifying selection (48, 49).

**TABLE 2:** Ranked list of gene clusters with intraclonal polymorphism, ranked by number of CTs affected by SNVs, short indels, or the presence or absence of gene polymorphisms within the CT

Cluster ID	Gene name(s)	Annotation	No. of CTs with^*a*^:							
			Intra-CT variation	Intra-CT gene presence variation	Short variants	Frame- shifts	Nonsense mutations	In-frame indels	Missense mutations	Synonymous mutations
**1164**	***lic2A***	**UDP-Gal–lipooligosaccharide** **galactosyltransferase**	**8**	0	11	8	0	2	1	0
**1473**	***hgpB***	**Hemoglobin-haptoglobin** **binding protein b**	**8**	0	161	5	0	3	68	85
1582	*hgpC*	Hemoglobin-haptoglobin binding protein C	**7**	4	118	0	0	2	54	62
0949	*ompP2*	Outer membrane protein P2 precursor	**6**	0	25	0	0	8	16	1
**1544**	***ompP1* (*fadL*)**	**Long-chain fatty acid** **outer membrane** **transporter**	**6**	1	40	4	3	0	20	13
**1182**	***tbp2***	**Transferrin-binding** **protein 2 precursor**	**5**	0	107	8	0	3	73	23
1488	*tuf*	Elongation factor Tu	**4**	6	7	0	0	0	2	5
1172	*spoT*	Guanosine-3',5'-bis-3'- pyrophosphohydrolase	**4**	0	5	0	0	0	4	1
1487	*group_1297*	Glycosyl transferase family 8 protein	**3**	1	3	2	0	1	0	0
1211	*hup*	Heme utilization protein Hup	**3**	0	76	0	0	1	31	44
1733	*group_438*	Putative uncharacterized protein	**3**	4	20	2	0	0	13	5
1441	*hsdM3*	Type I restriction enzyme HindVIIP M protein	**3**	0	77	2	0	2	11	62
1736	*hmw1C*, *hmw2C*	Putative glycosyltransferase involved in glycosylation of HMW1A and HMW2A	**3**	0	3	0	0	0	0	3
**1031**	***oafA***	**O-antigen acetylase**	**3**	0	3	3	0	0	0	0
1612	*hmw1A*, *hmw2A*	High-molecular-weight adhesin 1 or 2	**3**	1	5	0	0	0	5	0

a Variants occurring in different CTs were counted independently. Data are based on Data Sets S2 and S3. Boldface indicates clusters with high-impact short variants in three or more CTs.

10.1128/mBio.01176-18.10 DATA SET S3 Short variants found within CTs, based on alignment of short reads from all strains belonging to each of the 20 CTs with more than one isolate. Freebayes was used to identify an SNV, two or more SNVs in succession (MNV), and short-indel variants. Each tab reports on all short variants detected within each CT against the best (or earliest) assembly. The format (if exported as a tab-delimited file) conforms to a headerless variant call format (VCF) through the genotype columns. The next 4 columns report on the Roary cluster ID and annotation for liftover to the other CTs. The remaining columns are the results of the SNPEFF evaluation of variant impact. Splicing-related variants were ignored unless they also had additional impacts (format, .xlsx; 20 tabs, named by CT_reference ID). Download Data Set S3, XLSX file, 1.9 MB. Copyright © 2018 Moleres et al. 2018 Moleres et al. This content is distributed under the terms of the Creative Commons Attribution 4.0 International license.

### Independent loss-of-function mutations in *ompP1* or *fadL* arise within multiple lineages and patients.

**FIG 4:**
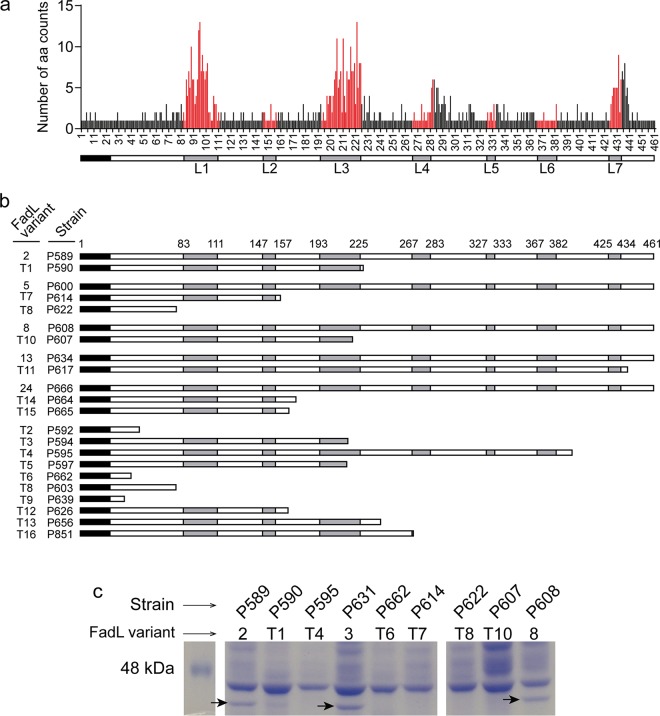
FadL amino acid variation and loss of alleles in COPD isolates. (a) Count of distinct amino acid variants at each position in an alignment of 34 full-length FadL proteins distributed among 68 isolates. Red positions correspond to residues in the seven predicted extracellular loops (L1 to L7). Nineteen unique full-length alleles were found in only one isolate; 15 were found in >1 isolate (with variant 5 being the most frequent). Thirteen CTs had only one full-length allele (CT3, -7, -16, -17, -18, -38, -45, -48, -54, -72, -73, -95, -137), and two had more than one (CT44 had -100). (b) Multiple independent *fadL* truncation alleles were observed and are schematically shown to indicate surface-exposed loops (in gray); they are paired with full-length alleles, when possible. (c) Putative null alleles do not express FadL protein. Whole-protein extracts from cultures were analyzed by SDS-PAGE and Coomassie brilliant blue staining. A protein band at FadL’s predicted molecular weight (arrows) was present in strains containing full-length *fadL* alleles (P589, P631, and P608; variants 2, 3, and 8, respectively) but absent in strains containing truncated alleles (P590, P595, P662, P614, P622, and P607; variants T1, T4, T6, T7, T8, and T10, respectively).

Aside from changes in phase-variable genes, the most striking observation of the parallel evolution analysis above was that of independent predicted loss-of-function changes in the *ompP1*, or *fadL*, gene (Fig. 1a; Fig. 4; Table 2; Data Set S2; Data Set S3). Seven of the 20 multi-isolate CTs were affected by polymorphisms in *fadL*, and this included nine independent null alleles induced by frameshift or nonsense mutations. Since only intra-CT polymorphisms were considered above and this was sometimes affected by annotation problems at truncated alleles, we extended the analysis by extracting the *fadL* allele from each of our new genome assemblies using tblastx (50). Multiple alignment of the resulting 92 amino acid sequences identified 34 distinct *fadL* alleles encoding a full-length protein, as well as 16 distinct alleles with mutations predicted to encode truncated proteins (Data Set S1b and e). Amino acid variation among the 34 full-length FadL protein variants is represented in Fig. 4a and shows high variability in predicted surface-exposed loops. Predicted full-length and truncated variants are illustrated in Fig. 4b and show clear evidence for multiple independent null mutations at different positions in the gene, including both frameshift and nonsense mutations. Truncation alleles were found in 24 isolates collected from 11 out of the 13 patients and spanning 14 CTs. Where possible, we identified full-length alleles that were the likely ancestors of specific truncated variants, when present in sets of isolates from the same patient and CT with no *fadL* variation outside the putative null mutation (groupings in Fig. 4b and Data Set S1e).

We further provide direct evidence that the FadL protein is not expressed in strains carrying predicted null alleles by SDS-PAGE of bacterial whole-cell lysates from representative isolates. A protein band whose weight was consistent with the FadL predicted molecular weight was present in strains P589, P631, and P608 but absent from P590, P595, P662, P614, P622, and P607 (Fig. 4c). Band identity as FadL was confirmed for strain P589 by peptide mass fingerprinting matrix-assisted laser desorption–ionization tandem time of flight (MALDI-TOF/TOF) mass spectrometry. Collectively, these results strongly suggest that *fadL* loss-of-function mutations are selected for during NTHi’s adaptation to the COPD lung.

### FadL variation affects hCEACAM1-dependent NTHi interactions with host cells.

**FIG 5:**
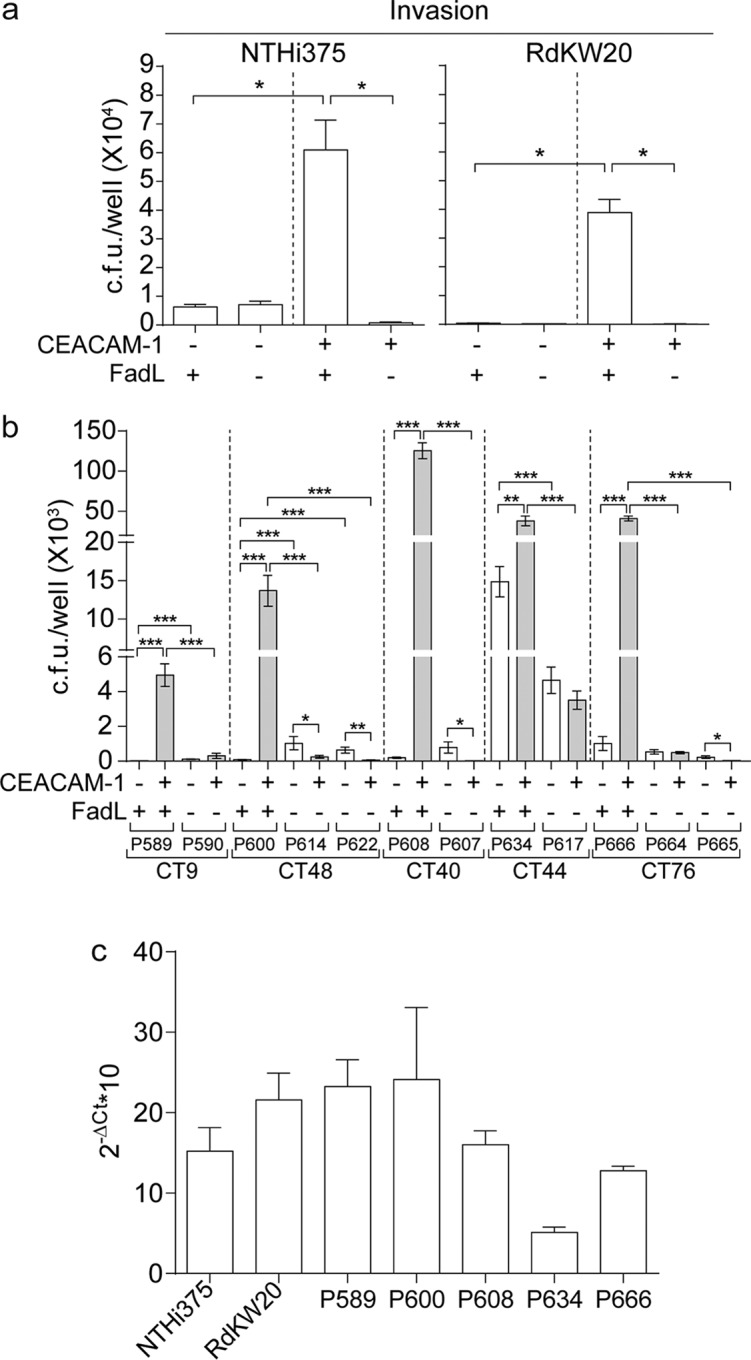
FadL_NTHi_ is a bacterial ligand of the hCEACAM1 receptor. (a) HeLa (nonexpressing hCEACAM1) and HeLa-BGP (a stably transfected derivative expressing hCEACAM1) cells were used to quantify epithelial invasion by WT and Δ*fadL* derivative NTHi375 and RdKW20. WT strains invaded HeLa-BGP cells at significantly higher rates than they invaded HeLa cells (for NTHi375 and RdKW20, *P* was <0.0001), in contrast to their respective Δ*fadL* mutants. Inactivation of *fadL* decreased the invasion of HeLa-BGP cells compared to that of their respective isogenic WT strains (*P* < 0.0001). (b) HeLa and HeLa-BGP cells were also used to quantify invasion by COPD patient isolates with the putative full-length FadL ancestor and derived truncated allele pairs (strains belonging to CT9, -48, -40, -44, and -76; FadL full-length variants 2, 5, 8, 13, and 24; FadL truncated variants T1, T7, T8, T10, T11, T14, and T15). Strains with full-length alleles invaded HeLa-BGP cells at significantly higher rates than they invaded HeLa cells (for P589, P600, P608, and P666, *P* was <0.0005; for P634, *P* was <0.005), in contrast to strains with truncated alleles. Moreover, *fadL* truncation alleles significantly decreased bacterial entry into HeLa-BGP cells compared to that of their full-length ancestors (*P* was <0.0005 when P590 was compared to P589; the *P* value was the same for comparisons of P614 and P600, P622 and P600, P607 and P608, P617 and P634, P664 and P666, and P665 and P666). (c) Expression of the *fadL* gene during exponential growth in sBHI as measured by quantitative reverse transcription-PCR. Data are shown for strains containing full-length FadL variants assayed in panels a and b, namely, NTHi375, RdKW20, P589, P600, P608, P634, and P666. In all cases, means ± standard deviations (SD) are shown. ΔCt, change in threshold cycle.

FadL in NTHi (FadL_NTHi_) strains has been reported to be a bacterial ligand for the human carcinoembryonic antigen-related cell adhesion molecule 1 (hCEACAM1), facilitating NTHi entry into epithelial cells (51, 52). Therefore, we assessed the impact of naturally occurring variation in *fadL* genes on the interplay between NTHi and host cells. By comparing bacterial invasion of HeLa cells (which do not normally express hCEACAM1) to that of a stably transfected HeLa derivative cell line expressing hCEACAM1 (HeLa-BGP cells) (53), we first tested whether bacterial cell entry depends upon the interaction between FadL and hCEACAM1. With isogenic *fadL* knockout mutations in reference strains NTHi375 and RdKW20 (54, 55), gentamicin protection assays showed strong defects in bacterial entry into HeLa cells in the absence of either FadL or hCEACAM1 in both strain backgrounds (Fig. 5a). As expected, COPD clinical isolates also showed FadL-dependent invasion of hCEACAM1-expressing cells. Pairs of clonal isolates carrying ancestral full-length and derived null alleles of FadL (Data Set S1e) were also tested. In all cases, invasion of hCEACAM1-expressing cells was substantially decreased in the absence of full-length FadL (Fig. 5b). Differences in hCEACAM1-dependent invasion rates were observed among isolates carrying distinct full-length FadL alleles, which could not be explained solely by differences in *fadL* gene expression levels; for example, isolate P608 showed the highest invasion rate, but its *fadL* gene expression was comparable to those of the other strains tested (Fig. 5c).

**FIG 6:**
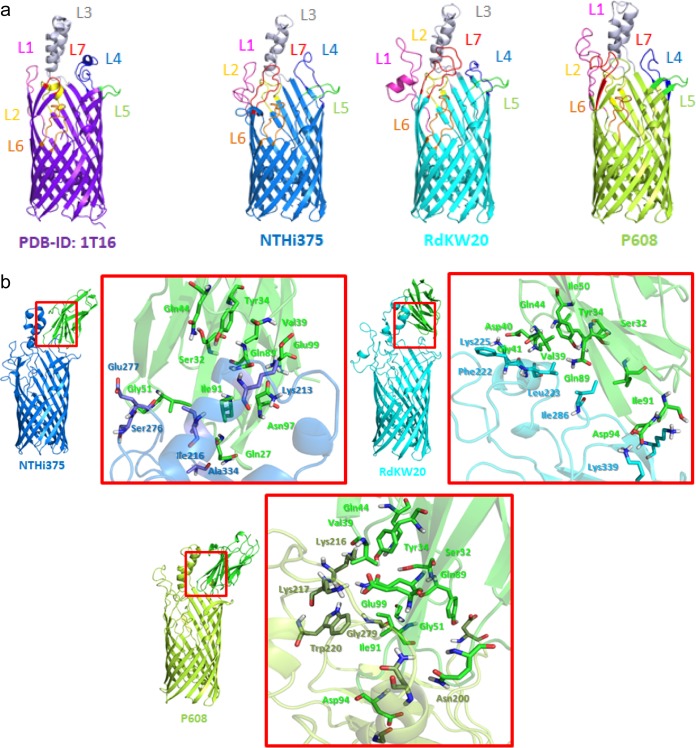
Structural modeling of the FadL interaction with hCEACAM1. (a) Computed homology models for H. influenzae FadL_NTHi375_ (in blue), FadL_RdKW20_ (in cyan), and FadL_P608_ (in lime green). The FadL_E. coli_ crystal structure is shown on the left (purple). The modeled structures correspond to a 14-stranded β-barrel with seven extracellular loops (L1 to L7), of which L1, L3, L4, and L7 are mobile and exposed to the outside. (b) Protein-protein docking between the amino-terminal domain of hCEACAM1 (green) and the modeled structures of FadL_NTHi375_ (blue), FadL_RdKW20_ (cyan), and FadL_P608_ (lime green). These interactions remained stable along molecular dynamics (MD) simulations of each hCEACAM1-FadL complex (Text S1).

We next examined the interaction of FadL_NTHi_ with hCEACAM1 by using computational molecular modeling, since a three-dimensional (3D) perspective of the molecular recognition events is lacking. To evaluate the potential effect of the natural variation present among the FadL surface-exposed loops, homology models of FadL variants from RdKW20, NTHi375, and P608 (selected as a representative COPD isolate with high rates of HeLa-BGP cell invasion) were computed using FadL in Escherichia coli (FadL_E. coli_) and refined by molecular dynamics (MD) simulations. All three computed 3D structures were similar to each other and to that of FadL_E. coli_, though the mobile variable surface-exposed loops had distinct conformations among structures, and some differences were seen within the β-barrel (Fig. 6a, Fig. S5a to c, and Text S1). Docking calculations (56) between the three FadL_NTHi_ models and hCEACAM1 were also performed and the resulting protein-protein complexes submitted to MD simulations, rendering high stability along the simulation. Protein-protein interaction analyses of the three FadL_NTHi_-hCEACAM1 complexes identified several relevant polar, hydrophobic, and CH-π interactions that remained stable along the MD simulation, which varied depending on the allele used (Fig. 6b, Fig. S5d, and Text S1). Among the residues involved in the interactions, we found several hCEACAM1 residues (Ser32, Tyr34, Val39, Gln44, Gln89, and Ile91) that were previously reported as necessary for the interaction with NTHi by site-directed mutational analyses (57, 58). Our structural modeling clearly identifies these residues as directly involved in the interaction with FadL, thereby accounting for the interaction between FadL_NTHi_ and hCEACAM1 and further suggesting that natural variation in FadL surface-exposed loops may affect NTHi’s ability to interact with epithelial cell surfaces. Our molecular modeling calculations also allowed us to identify several FadL residues located in loops 3, 4, and 5 as likely to be involved in hCEACAM1 binding. High conservation among FadL full-length allelic variants possibly excludes many of these from an association with variability in epithelial invasion. In particular, Gly193_P608_ in L3, Ile286_RdKW20_ and Lys290_RdKW20_ in L4, and Asp332_P608_, Ala334_NTHi375_, and Lys339_RdKW20_ in L5 are highly conserved. However, other identified residues are polymorphic, in particular, Val196_P608_, Asn200_P608_, Lys213_NTHi375_, Leu214_P608_, Lys216_P608_, Lys217_P608_, Trp220_P608_, Phe222_RdKW20_, Lys225_RdKW20_, Ser276_NTHi375_, and Gly279_P608_. These variants may account for the observed variation in terms of epithelial invasion by the tested strains.

10.1128/mBio.01176-18.1 TEXT S1 Additional detailed bioinformatics and experimental methods, as well as supplementary results related to the protein structural modeling work. Download Text S1, DOCX file, 0.1 MB. Copyright © 2018 Moleres et al. 2018 Moleres et al. This content is distributed under the terms of the Creative Commons Attribution 4.0 International license.

10.1128/mBio.01176-18.6 FIG S5 (a) Superimposition of the three homology-based models (FadL_RdKW20_ is in cyan, FadL_NTHi375_ is in blue, and FadL_P608_ is in lime green) and the X-ray crystal structure from FadL_E. coli_ (PDB accession number 1T16, in purple). Main different and common amino acids in the inner β-barrel are shown as sticks. Regarding inside the β-barrel, species-specific differences in specific residues were identified. At FadL_E. coli_ position Ala319, Lys is found in the FadL models; in FadL_E. coli_ positions Phe33 and Phe346, Val and Tyr are, respectively, found in the FadL models; in FadL_E. coli_ position His108, there is Tyr; in FadL_E. coli_ position Glu344, there is Leu; in FadL_E. coli_ position Lys347, there is Ser; and in FadL_E. coli_ position Ala349, there is Asn in the FadL models. Regarding the loops, some differences were found in the predicted 3D folding and, consequently, in their mobility along the molecular dynamic (MD) simulations. In particular, loops L1, L3, L4, and L7 exhibited high mobility along the MD. (b) Root mean square deviation (RMSD) of the backbone of the different models of FadL on the left, and root mean square fluctuation (RMSF) of the backbone of FadL_NTHi375_ (blue), FadL_RdKW20_ (cyan), and FadL_P608_ (green) on the right. (c) Three different conformations of FadL_NTHi375_ (blue, left), FadL_RdKW20_ (cyan, middle), and FadL_P608_ (green, right) from normal-mode analysis (NMA) are superimposed. (D) RMSD of the backbone of FadL_NTHi375_ (blue) with hCEACAM1 (green) on the left, FadL_RdKW20_ (cyan) and hCEACAM1 (green) in the middle, and FadL_P608_ (dark green) and hCEACAM1 (green) on the right. Download FIG S5, PDF file, 1.2 MB. Copyright © 2018 Moleres et al. 2018 Moleres et al. This content is distributed under the terms of the Creative Commons Attribution 4.0 International license.

### FadL loss-of-function confers resistance to the bactericidal effect of arachidonic acid.

**FIG 7:**
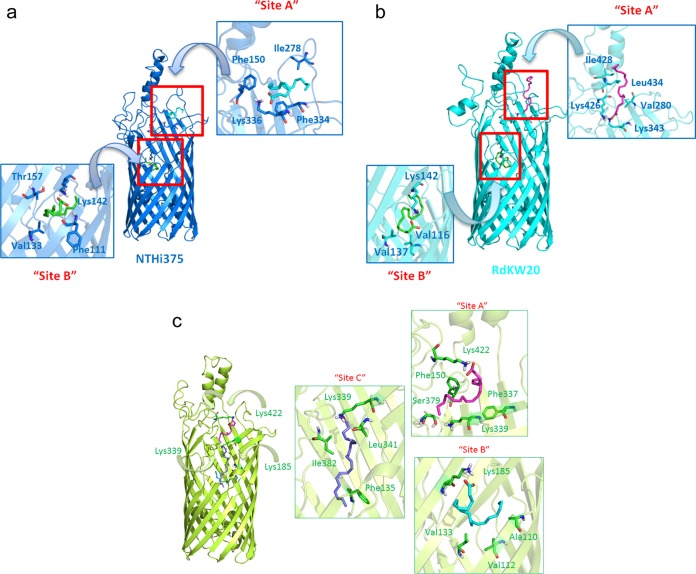
Docked binding poses of arachidonic acid (AA) in FadL_NTHi_. Docked binding poses of AA are shown for FadL_NTHi375_ (blue) (a), FadL_RdKW20_ (cyan) (b), and FadL_P608_ (lime green) (c). The details of the interactions are depicted as sticks. Calculations predicted two favorable binding modes of AA in both structures (FadL_NTHi375_ and FadL_RdKW20_); the first one was at the entrance of the β-barrel (site A) in a hydrophobic groove located between L3 and L4. Interactions with polar residues, specifically with Lys at L5 (Lys336 in FadL_NTHi375_ and Lys426 in FadL_RdKW20_), lipophilic interactions, and CH-π interactions with aliphatic and aromatic residues were identified; and a second binding mode at the depth of the β-barrel (site B), establishing polar contact with Lys142 present in L2. These binding poses were also found for FadL_P608_ together with an additional binding site (Text S1). Details of the interactions are represented as sticks.

FadL_NTHi_ is also a predicted transporter of exogenous long-chain fatty acids (LCFAs). In E. coli and Sinorhizobium meliloti, the *fadL* gene products are required for the ability to use free fatty acids as a sole carbon source (59–61), and key amino acid residues required for fatty acid binding and lateral diffusion have been elucidated for FadL_E. coli_ (59, 61). To investigate the ability of FadL_NTHi_ to bind LCFAs, we performed docking calculations of three LCFAs in the FadL_NTHi375_, FadL_RdKW20_, and FadL_P608_ homology models. To take into account the flexibility of FadL, additional normal mode analysis (NMA) of the three FadL_NTHi_ 3D models provided distinct conformations representing motions and conformational changes. Tested LCFAs included arachidonic acid (AA), oleic acid (OA), and lauryl dimethylamine-*N*-oxide (LDA) as a control (since it was previously crystallized with FadL_E. coli_). All the calculations predicted several binding poses for the three fatty acids at the entrance to and within the FadL_NTHi_ β-barrel (Fig. 7 and Fig. S6). All three fatty acids were predicted to interact with FadL_NTHi_ at sites equivalent to those for LDA in FadL_E. coli_, but other distinct binding sites were also identified inside the β-barrel (Text S1). The docking calculations provide reasonable binding poses to FadL_NTHi_ for the studied LCFAs and support its role as a fatty acid transporter. However, further use of LCFAs by NTHi as a sole carbon source was deemed unlikely due to the absence of a complete β-oxidation pathway in NTHi (26, 54). Thus, as expected, no bacterial growth was observed in a defined minimal medium free of fatty acids when supplemented with AA as a carbon source (62).

10.1128/mBio.01176-18.7 FIG S6 (a) Docked binding poses of lauryl dimethylamine-*N*-oxide (LDA) in FadL_NTHi375_ (blue) on the left, FadL_RdKW20_ (cyan) in the middle, and FadL_P608_ (lime green) on the right. For the FadL_NTHi375_ model, LDA is predicted to bind inside sites A and B. In the case of the FadL_RdKW20_ model, LDA is predicted to bind inside sites B and C. For the FadL_P608_ model, LDA is predicted to bind to the three sites A, B, and C. (b) Docked binding poses of oleic acid (OA) in FadL_NTHi375_ (blue) on the left, FadL_RdKW20_ (cyan) in the middle, and FadL_P608_ (lime green) on the right. In the case of the FadL_NTHi375_ model and FadL_RdKW20_ models, OA is predicted to bind inside sites A and B. For the FadL_P608_ model, OA is predicted to bind to the three sites A, B, and C. Download FIG S6, PDF file, 0.9 MB. Copyright © 2018 Moleres et al. 2018 Moleres et al. This content is distributed under the terms of the Creative Commons Attribution 4.0 International license.

**FIG 8:**
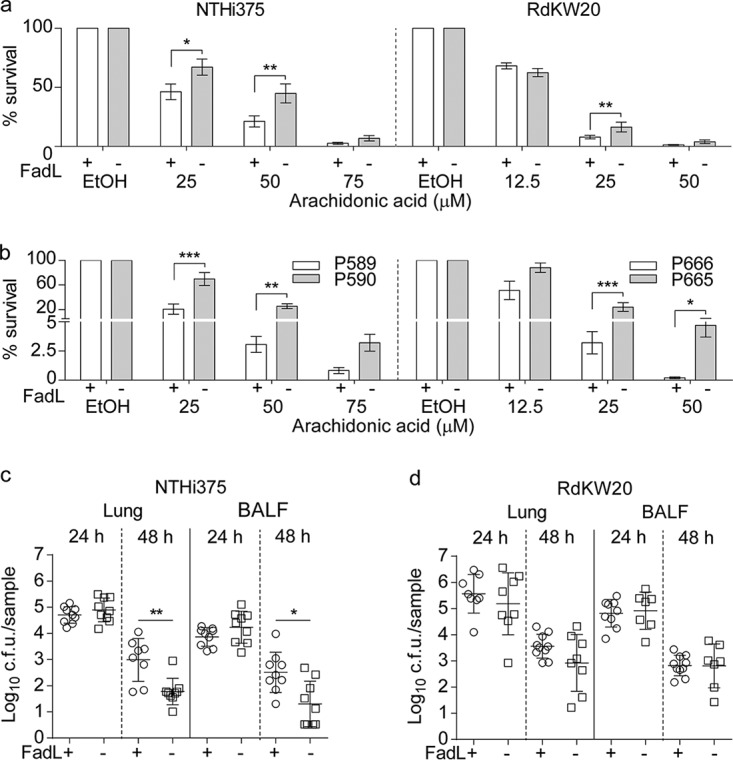
FadL loss of function confers resistance to arachidonic acid. (a) A dose-dependent bactericidal effect of AA was observed for both WT NTHi375 and WT RdKW20. Both *fadL* mutants were more resistant to AA than their respective parental strains (NTHi375 with AA at 25 μM, *P* < 0.01; AA at 50 μM, *P* < 0.005; RdKW20 with AA at 25 μM, *P* < 0.005). EtOH, ethyl alcohol. (b) FadL natural loss of function increases NTHi resistance to AA. Fatty acid resistance was assayed for representative clonal isolate pairs containing two predicted FadL full-length ancestor and derived truncated allele pairs. Strains with FadL truncations (P590 and P665) were more resistant to AA than their respective clonal counterparts (P589 and P666, *P* < 0.05 for both pairs at 25 and 50 μM). (a and b) Results are expressed as percentages of bacterial survival (means ± standard errors) compared to survival with the use of a vehicle solution. (c and d) Inactivation of the *fadL* gene has little effect in a murine lung acute-infection model. CD1 mice were intranasally infected with WT (circles) or Δ*fadL* (squares) strains. Mice were euthanized, and bacterial loads in lungs (left) and bronchoalveolar lavage fluid (BALF) (right) samples (log_10_ numbers of CFU/sample) were quantified. Data are shown as means ± SD for the NTHi375 (c) and RdKW20 (d) backgrounds. No differences among strains were observed at 24 hpi. At 48 hpi, no significant changes were observed for the RdKW20 strain; in NTHi375, *fadL* inactivation weakly reduced bacterial loads (*P* < 0.005 in lung; *P* < 0.01 in BALF).

Free fatty acids act as natural detergents, and a bactericidal effect on H. influenzae has been described for AA (63). We next examined the viability of wild-type (WT) and isogenic *fadL* mutant strains when they were incubated in defined minimal medium with AA. WT strains had reduced viability in a dose-dependent manner; by contrast, both *fadL* knockout mutants were more resistant to AA than their respective WT strains (Fig. 8a). The same effect was seen for OA, though only at higher concentrations (data not shown). Resistance to AA was also assayed for two representative pairs of COPD isolates with predicted ancestral full-length and derived null alleles of FadL (Data Set S1e). In both cases, loss of FadL function was associated with increased bacterial resistance to AA (Fig. 8b). Although one or more of the few additional genetic variants that distinguish the two pairs of isolates may instead be responsible for this increased AA resistance, *fadL* loss of function was the only common one in both isolate pairs (Table 3). Given that AA metabolites are key players in COPD-related airway inflammation (64), these molecules may in turn provide a selective pressure within COPD patient lungs such that NTHi *fadL* loss-of-function mutations are adaptive within this niche.

**TABLE 3:** Coding region SNVs in CT9 strains P590 and P589 and CT76 strains P665 and P666^*a*^

CT	Ref nt	Alt nt	Mutation type	Impact	Gene name	Ref aa	Alt aa	aa position	Protein length	Function
CT9	A	C	Missense	Moderate	L_00407	Ser	Arg	22	470	Putative ATPase, hypothetical protein
	A	C	Missense	Moderate	L_00407	Asn	Thr	282	470	Putative ATPase, hypothetical protein
	G	T	Missense	Moderate	L_00640	Pro	Thr	513	844	Translation initiation factor IF-2
	A	C	Stop lost	High	L_00728	Ter	Ser	229	228	Outer membrane protein P1, long-chain fatty acid outer membrane transporter
	T	C	Missense	Moderate	L_01000	Asn	Asp	59	137	Phage GP46 family protein, hypothetical protein
	A	G	Missense	Moderate	L_01011	Ser	Pro	91	101	Putative uncharacterized protein, putative protein
	C	A	Missense	Moderate	L_01014	Glu	Asp	131	161	Phage virion morphogenesis protein
	T	C	Missense	Moderate	L_01016	Asn	Asp	521	546	Putative uncharacterized protein, hypothetical protein
	TG	CA	Missense	Moderate	L_01016	Lys	Glu	514	546	Putative uncharacterized protein, hypothetical protein
										
CT76	AGGA	AA	Frameshift	High	L_00010	Gly	FS	140	258	Hypothetical protein
	A	C	Missense	Moderate	L_00404	Lys	Thr	413	1046	Outer membrane receptor protein, putative Fe transport, hemoglobin/ hemoglobin-haptoglobin binding protein B
	CT	CTCGAT	Frameshift and stop lost	High	L_00626	Ter	FS	168	167	Outer membrane protein P1, long-chain fatty acid outer membrane transporter
	T	C	Noncoding transcript	Modifier	L_00890					rRNA
	A	C	Missense	Moderate	L_00973	Tyr	Asp	345	366	Outer membrane protein P2 precursor
	C	T	Missense	Moderate	L_00973	Gly	Ser	93	366	Outer membrane protein P2 precursor
	A	C	Missense	Moderate	L_01164	Gln	Pro	288	704	Guanosine-3',5'-bis 3'- pyrophosphohydrolase
	C	T	Missense	Moderate	L_01164	Pro	Leu	402	704	Guanosine-3',5'-bis 3'- pyrophosphohydrolase
	T	A	Noncoding transcript	Modifier	L_01171					rRNA
	A	T	Noncoding transcript	Modifier	L_01171					rRNA
	T	G	Missense	Moderate	L_01757	Ser	Ala	322	366	Histidinol-phosphate aminotransferase 2

a nt, nucleotide; Ref, reference; Alt, alternative; aa, amino acid; FS, frameshift. The CT9 strain P589 contained each SNV; P590 contained none of them, it was used as reference. CT76 strain P666 contained each SNV; P665 contained none of them, it was used as reference.

### FadL inactivation has no effect in a murine model of acute lung infection.

Human-restricted bacterial pathogens are known to selectively interact with hCEACAM1 (65). NTHi murine pulmonary infection may uncouple FadL functional analysis as an LCFA transporter and a hCEACAM1 ligand, since the latter is absent from mice. So, CD1 mice were infected with WT and *fadL* mutant NTHi375 and RdKW20 strains, and bacterial loads were quantified in lung and bronchoalveolar lavage fluid (BALF) samples. At 24 h postinfection (hpi), bacterial counts were comparable for both WT and mutant strain pairs. At 48 hpi, no differences were observed for WT and mutant RdKW20 strains; in contrast, NTHi375Δ*fadL* rendered lower bacterial counts than its isogenic WT strain (*P* < 0.005 in lung, *P* < 0.01 in BALF) (Fig. 8c and d). Together, these results showed that *fadL* gene inactivation gives no substantial benefit in this infection model. We acknowledge that this model system may be suboptimal in this case, since NTHi fails to establish a long-term infection, showing only variation in times to clearance, which occurs in <3 days for the WT strains.

### Truncated *fadL* alleles appear to arise in NTHi within COPD patient lower airways and are rare in isolates from other body sites.

Because all the subjects in this study had COPD for years prior to being sampled for NTHi, we could not conclude that null alleles of *fadL* arose within COPD patient lungs, despite 6 of 20 multi-isolate CTs being polymorphic for null alleles, since these might also arise in the normal commensal population of NTHi in the nasopharynx. To test whether loss of *fadL* function was specifically associated with lower airway infections, we first examined the distribution of full-length and truncated *fadL* alleles across 181 publicly available genomes (excluding those from the Pettigrew et al. study [36]) derived from isolates with diverse clinical origins (26, 66, 67) (Fig. S4). Of 153 isolates collected outside the context of lower respiratory tract infection (including nasopharyngeal carriage, middle ear carriage in pediatric patients with otitis media, invasive bacteremia, and meningitis isolates), only three non-lung isolates’ genomes lacked a single full-length *fadL* annotation (1.9%, one from a patient with otitis media, one from a patient with bacteremia, and one other). In contrast, out of 28 isolates from the lower airways (isolates from COPD patient sputum or other pulmonary infections), 9 had truncated *fadL* genes (33.3%). This demonstrates that an intact *fadL* gene is the norm among NTHi strains as a whole, but loss-of-function mutations are significantly enriched in lung isolates (Fisher’s exact test *P* value = 1.3 × 10^−6^).

As we were completing this study, 269 new NTHi genomes collected from American COPD patients were published by Pettigrew et al. (36), providing us with a second independent test of the hypothesis that *fadL* null mutations are selected for during long-term COPD-associated infections. Indeed, 43 of all 269 genomes (16.0%) had truncated/absent *fadL* genes, as determined by Roary gene clustering analysis. Aggregating across assemblies gave consistent results: only 3 of 159 nonlung isolates lacked a predicted full-length *fadL* gene, whereas 69 of 399 lung/sputum isolates lacked it (1.9% versus 17.3%, Fisher’s exact test *P* value = 4.2 × 10^−8^).

A comparison of the frequencies of truncated *fadL* between the persistent and cleared isolates in the Pettigrew et al. data set found a weak (but not significant) trend toward higher rates of truncated *fadL* alleles in persistent isolates than in cleared isolates (37 of 202 persistent strains versus 6 of 67 cleared strains; odds ratio, 2.2; Fisher’s exact test *P* value = 0.084). Finally, because persistent isolates had been carefully chosen to pair the first and last isolates with matched MLSTs from the same patient, we also asked whether paired isolates tended to show loss or gain of *fadL* function over time. Of 101 MLST-matched pairs, 11 were polymorphic for *fadL* between the first and last isolation dates, and all 11 had a full-length allele at the first visit and a truncated allele at the last visit (binomial test *P* value = 0.0001), providing strong evidence that *fadL* truncation mutations arise mostly *in situ* in the COPD lung.

Taken together, our results strongly suggest that intact *fadL* function is normally beneficial for H. influenzae but becomes deleterious in the context of lower respiratory tract infections, likely due to the *fadL*-dependent bactericidal effect of LCFAs being upregulated in COPD patient lungs.

## DISCUSSION

Our comparative genomic analyses of longitudinally collected NTHi isolates from COPD patients provides novel insights into the evolutionary dynamics of a bacterium as it adapts to becoming a chronically infecting lung pathogen. This work and that of Pettigrew et al. (36) present data from two independent cohorts (Spanish and U.S. patients, respectively), and both identify an overlapping set of NTHi genes that change during long-term COPD infection. COPD strains show no phylogenetic clustering based on clinical source, geography, duration of persistence, or year of isolation, and phase-variable genes were a frequent source of genetic variation among clonally related isolates. Beyond the major findings in the work of Pettigrew et al. (36), we identified probable recombination and interpatient transmission events, as well as provided experimental evidence of the molecular mechanism behind selection for COPD-specific recurrent loss-of-function mutations in the NTHi gene *ompP1*, or *fadL*.

This study was motivated by an earlier one in which we conducted a limited genomic analysis of three serially collected NTHi isolates belonging to a single CT recovered from a single bronchiectasis patient, which showed increasing resistance to antimicrobial peptides and also carried amino acid substitutions in the SapABCDFZ system, which confers antimicrobial peptide resistance (68). Here, we expanded to a larger set of longitudinally collected COPD isolates. Notably, we identified intraclonal polymorphisms from 20 multi-isolate CTs collected over time, rather than in only the first and last in each patient series (36). We found evidence for parallel evolution in several NTHi genes, in which multiple CTs carried coding polymorphisms in the same genes, suggestive of *in situ* natural selection. In particular, the *ompP1*or *fadL* locus stood out because of the high frequency of independent loss-of-function alleles seen in different CTs and in different patients. Our observations are consistent with those of other studies that have identified amino acid variation in the FadL protein among H. influenzae and Neisseria meningitidis strains (51, 69, 70) and are congruent with reports on the key role of outer membrane proteins for within-host adaptive strategies (71, 72).

The recurrent null mutations seen in *fadL* within NTHi CTs isolated from COPD patient sputum samples suggest a strong selective pressure to lose this gene function during adaptation to the COPD lung, consistent with NTHi’s reductive evolution toward becoming a long-term resident of the lower airways (73). In support of this, our survey of >450 NTHi genomes found that null mutations in *fadL* were extremely rare except in isolates from lung infections. Given that the FadL_NTHi_-hCEACAM1 interaction was known (51) and strongly supported by our computational 3D models, we were initially puzzled how losing this interaction could be beneficial, but finding that loss of FadL also confers resistance to microbicidal fatty acids suggests antagonistic pleiotropy, wherein a single gene affects multiple phenotypes, each of which may be beneficial or deleterious depending on conditions. Antagonistic pleiotropy can be modulated by gene inactivation, such that loss-of-function mutants can outcompete the WT parental strains once a trait becomes deleterious (74). This may be the case for FadL: loss of the functional gene avoids triggering bacterial cell death by the combined fatty acid detergent effect and NTHi’s inability to metabolize these types of molecules. The molecular basis for the fatty acid detergent effect on NTHi viability is unknown. Of note, Helicobacter pylori has features common to NTHi’s susceptibility to free fatty acids, the lack of a β-oxidation pathway, and the presence of an active natural competence pathway (75–78). Despite occupying separate niches of the human body, both pathogens might have common adaptive strategies contributing to colonization linked to chronic disease conditions. Other explanations for recurrent *fadL* loss-of-function mutations are plausible given that, as a surface protein, FadL may be subject to immune pressure and so bacteria may benefit from its loss.

**FIG 9:**
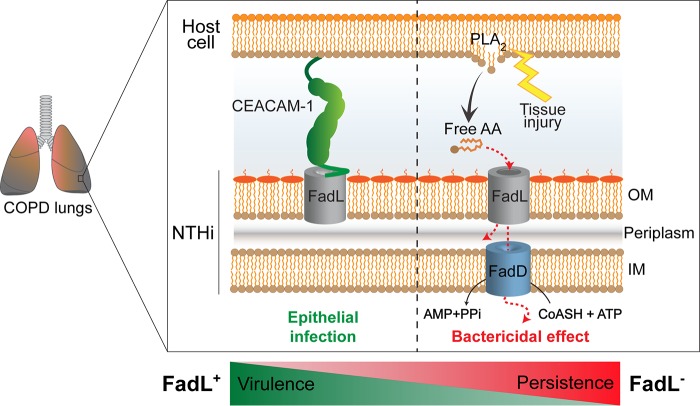
Model illustrating FadL, or OmpP1, antagonistic pleiotropy during H. influenzae adaptation in COPD patient lungs. FadL, or OmpP1, is a bifunctional outer membrane protein that binds both the hCEACAM1 receptor (left panel, green) and free long-chain fatty acids (LCFAs) (right panel, red). This study provides evidence for recurrent *fadL* mutations being a likely case of antagonistic pleiotropy associated with bacterial pathoadaptation in COPD patient lungs. In particular, loss of FadL function reduces NTHi’s ability to infect epithelia but also increases its survival in the presence of bactericidal LCFAs, which are inflammatory markers enriched within the COPD lung. FadL-hCEACAM1 binding may be essential for virulence, but its persistence in the lower airways may indeed be favored by the inability of FadL to, thus, overcome free-LCFA selective pressure. OM, outer membrane; IM, inner membrane; CoASH, coenzyme A.

Several lipids are molecular mediators of respiratory diseases (79). In particular, AA metabolites are key players in COPD airway inflammation (64). Phospholipase A_2_ (PLA_2_)-catalyzed hydrolysis of membrane phospholipids results in production of free fatty acids, most importantly AA, which serves as a precursor for inflammatory mediators, such as platelet-activating factor (PAF) (80). In turn, lipoprotein-associated PLA_2_ mediates PAF hydrolysis and dampens PAF-mediated inflammation (81). H. influenzae exploits molecular mimicry to evade host inflammation through GlpQ, an enzyme that hydrolyzes phosphorylcholine (ChoP) moieties shared by both PAF and the bacterial LOS molecule, therefore removing PAF from the airways and suppressing inflammation. Such bacterially driven regulation of PAF signaling might be overcome by a therapeutic increase of PAF levels at the site of infection through PLA_2_ targeting (82), which in turn, might also dampen free AA release in COPD patient lungs, therefore limiting selection against FadL function during NTHi pathoadaptation (for a model, see Fig. 9).

Finally, we acknowledge that our strain collection presented some limitations. First, we are unlikely to have sampled the initial colonizing bacterial isolates; therefore, we did not order the observed genetic variations or determine the most recent common ancestor (13). Second, we analyzed single isolates from each sampling point, yet we know that NTHi colonization and infection are often polyclonal (24, 32, 37–40). Despite these considerations, our results show the utility of comparing the genomes of bacterial isolates collected over time from long-term chronic infections, showing that genetic polymorphisms within clonally related NTHi isolates can help tease apart transmission of strains between subjects and the genetic changes that result in changes in pathogenic traits. Here, only a small number of genes showed recurrent mutations in different bacterial CTs, and our follow-up studies support a bifunctional role for FadL in both interactions with host epithelia and interactions with free fatty acids.

We conclude that the environment of the COPD patient lung exerts a specific selective pressure to lose functional *fadL*, and this is likely by providing resistance to bactericidal fatty acids, in spite of gene truncation also compromising the ability of NTHi to invade epithelial surfaces. We speculate that FadL-hCEACAM1 binding may be essential for infection establishment but that, once established, persistence may favor the loss of FadL to overcome free fatty acids pressure. This hypothesis aligns with the observed low hCEACAM1 expression in lung tissue (83). Lastly, we highlight PLA_2_ as a potential target for host-directed therapeutics against NTHi chronicity in the setting of COPD.

## MATERIALS AND METHODS

### COPD strain isolation and PFGE typing.

H. influenzae was identified by culture methods (84) and with MALDI-TOF Biotyper, version 3.0 (Bruker). NTHi isolates were genotyped using PFGE (85).

### Bacterial growth conditions and genetic manipulations.

H. influenzae COPD isolates (Data Set S1a) and reference strains RdKW20 and NTHi375 were grown at 37°C with 5% CO_2_ on chocolate agar (bioMérieux) or supplemented brain heart infusion (sBHI), made from BHI supplemented with 10 μg/ml hemin and 10 μg/ml NAD, with or without agar for solid and liquid culture, respectively. Spectinomycin 30 μg/ml (Spec_30_) was used when needed. E. coli was grown on Luria Bertani (LB) broth, with or without agar, at 30°C or 37°C and supplemented with ampicillin at 100 μg/ml (Amp_100_) or Spec_50_, as needed. The *fadL* gene was knocked out by replacement of the coding sequence with a spectinomycin-resistant (Spec^r^) cassette as described previously (86) (see Text S1 in the supplemental material and Table 4).

**TABLE 4:** Primers used in and generated by this study

Primer	Sequence (5′–3′)
fadL-qPCR-F1/1605	CTACTTCTGGGCTTGGTCGT
fadL-qPCR-F2/1606	CTACTTCTGGGCTTGGCCGT
fadL-qPCR-F3/1658	CTACTTCAGGGCTTGGTCGT
fadL-qPCR-R1/1607	GCAACTAACCCAGCTTTGATGA
fadL-qPCR-R2/1608	GCAACAAACCCGGCTTTAATGA
fadL-F1/1302	GTTTTCAACTGCCACGATTTCGCT
fadL-R1/1303	CCCACGGCACGAACAGCTTTAGGA
fadL-F2/1358	TGTCGTGGCAACTAACCCAGCTTTGATGAGTTTATTTAAAACGGCACAGTATTCCGGGGATCCGTCGACC
fadL-R2/1359	TCAAGATTATAACTTGCCCCTAATGCAACACGAGAGTTATTACTGTATTGTGTAGGCTGGAGCTGCTTCG
gyrA-qPCR-F/1078	ATATGTTGGTTGATGGGCAAGG
gyrA-qPCR-R/1079	GGCGAGAAATTGACGGTTTCT

### Genome sequencing, assembly, annotation, and data deposition.

DNA was extracted from each isolate and sequenced using standard procedures (Text S1). All 92 genomic DNAs were subjected to Illumina paired-end short-read sequencing, and a subset of 17 DNAs were also subjected to Pacific Biosciences RSII long-read sequencing to generate covalently closed circular genomes. Genome assembly was platform specific (Text S1). Assemblies were taxonomically classified with Taxator-tk v1.2 (87). For MLST strain identification, the *adk*, *atpG*, *frdB*, *fucK*, *mdh*, *pgi*, and *recA* genes were extracted from assemblies and assigned STs (https://github.com/tseemann/mlst). New alleles and STs were submitted as needed (Data Set S1b; https://pubmlst.org/hinfluenzae). To compare PacBio and Illumina assemblies of the same isolates’ genomes, progressiveMauve (snapshot_2015-02-25) (88) was used to reorder Illumina contigs against the complete PacBio assembly, and perfect agreement was found in all cases. To annotate strains with consistent gene names, a database of annotated *Pasteurellaceae* genomes from the NCBI database was created (https://github.com/rehrlich/prokka_database_maker). Assemblies were annotated using this database and Prokka (v1.11) (89). All raw reads and assemblies were deposited in NCBI under BioProject number PRJ282520; BioSample accession numbers for each strain are included in Data Set S1b and c. Publicly available genome assemblies at the NCBI and the Sanger Centre were downloaded (*n* = 572) and reannotated with Prokka to maintain consistency. Assemblies were used only if they had <50 contigs, except for the Pettigrew et al. genomes (BioProject accession number PRJNA358390), where assemblies with <100 contigs were retained (except that all 269 assemblies were used in the *fadL* truncation analysis). This combined data set of 391 publicly available assemblies and 92 assemblies sequenced for this study were used for gene clustering, phylogenetic analysis, and clonal typing.

### Gene clustering, phylogenetic analysis, and clonal typing.

Homologous protein-coding genes among all genomes were clustered together using Roary at a BLASTp threshold of 75% (90). Phylogenetic tree reconstruction used the hybrid MPI/Pthreads version of RAxML (8.2.4, 10 processes, 16 central processing units [cpu] per process, -f a -x 1234 -m GTRGAMMA -p 7 -N autoMRE -j set thread affinity) on a concatenation of all codon-aware alignments of protein-coding genes with at most one copy per strain (91). PFGE and MLST are low-resolution strain typing methods, so we applied goeBurst via PHYLOViZ 2.0 (92) to cluster isolates into CTs, based on their allelic identity across 309 core protein-coding genes shared by all H. influenzae isolates studied here, as well as several H. haemolyticus and H. parainfluenzae strains. CTs were defined as sets of strains differing by no more than 15 allelic differences in the core gene set.

### Detecting intraclonal genetic variation in protein-coding genes.

Because methods to call genetic variants by short-read alignment to reference assemblies are relatively mature compared to methods that use whole-genome alignment, we chose a reference sequence from each CT (based on assembly quality, a Pacific Biosciences assembly when available) and then aligned short-read pairs from all isolates belonging to that CT to this reference (Burrows-Wheeler Aligner [bwa] mem version 0.7.5a-r405). Single-nucleotide and short-insertion/deletion variants (SNVs and indels, respectively) were extracted using freebayes (v1.1.0) (filtering out variants with a quality score of <1), and the impact of coding variants was evaluated using SnpEff (v4.3k) (93). To identify homologs across CTs, the LiftOver tool was used on the Roary gene possession table described above to determine gene identities between the CT reference genomes. Artifacts of gene annotation failure (for example, due to frameshift and nonsense mutations), particularly for the CT references, were reconciled by considering intraclonal variation in gene possession as a high-impact variant.

### FadL allelic identities for whole collection.

To extract the *fadL* gene sequence from each of the new NTHi genomes independently of the Prokka annotation, a local database containing the 92 whole-genome sequences was created and the local BLAST tool was employed (94). Best hits to *fadL* were extracted from each genome using bedtools (95), followed by translation and alignment using Clustal Omega.

### Protein and gene expression analysis.

Whole-protein extracts from bacterial cultures were analyzed on 12% SDS-PAGE 20- by 20-cm gels designed for long-range protein separation with maximum resolution under reducing conditions (Bio-Rad, PROTEAN II xi Cell), followed by Coomassie brilliant blue staining. Reverse transcriptase real-time quantitative PCR used total RNA extractions from exponentially grown bacterial cultures (optical density at 600 nm [OD_600_] = 0.6), with primers for *fadL* and primers for *gyrA* as endogenous controls (Table 4).

### Cell culture and bacterial infection.

HeLa (ATCC CCL-2) and HeLa-BGP (kindly provided by Alain Servin, Université Paris-Sud, France) (53) cells were seeded (4 × 10^5^ cells/well 24 h before infection) and invasion assays performed as described previously (96).

### Free fatty acid susceptibility testing.

Bacterial suspensions were incubated with various concentrations of free fatty acids or vehicle solution (ethanol), serially diluted, and plated on sBHI agar. Results are expressed as percentages of bacterial survival ([CFU ml^−1^ of LCFA/CFU ml^−1^ of the vehicle solution] × 100).

### NTHi mouse lung infection.

A CD1 mouse model of NTHi pulmonary infection was used (97). At least five mice per treatment (genotype by time point) were intranasally inoculated with ∼2 × 10^8^ CFU/mouse. Mice were euthanized at 24 or 48 hpi. Total log_10_ numbers of CFU in lung and BALF samples per mouse were determined.

### Computational modeling.

Homology modeling structures of the three FadL_NTHi_ amino acid sequences were obtained from SWISS-MODEL using FadL_E. coli_ (PDB accession number 1T16) (98), and subsequent MD simulations were performed with AMBER14 (98). Structure refinement of hCEACAM1 (PDB accession number 4QXW) was performed by means of MD simulations. Protein-protein docking of the three FadL_NTHi_ structures with hCEACAM1 was performed with ZDOCK (56). LCFA docking calculations were performed with AutoDock 4.2.2 (99). Detailed protocols for the calculations can be found in Text S1.

### Ethics approval and consent to participate.

NTHi isolates were collected at the Hospital Universitari Bellvitge (HUB), a tertiary-care hospital in Barcelona, Spain. NTHi strains were isolated from good-quality sputum samples of 13 COPD patients over a 14-year period (2000 to 2014) at regular check-ups or during visits requiring hospitalization due to an exacerbation of the disease. Informed consent was not required, as sputum sampling and the microbial isolation process are part of the standard microbiological routine. Patient confidentiality was protected in all cases. For animal experiments, CD1 female mice aged 4 to 5 weeks were purchased from Charles River Laboratories (France) and housed under pathogen-free conditions at the Institute of Agrobiotechnology facilities (registration number ES/31-2016-000002-CR-SU-US). Animal handling and procedures were in accordance with the current European (Directive 86/609/EEC) and National (Real Decreto 53/2013) legislations, according to the FELASA and ARRIVE guidelines, and with the approval of the Universidad Pública de Navarra (UPNa) Animal Experimentation Committee (Comité de Ética, Experimentación Animal y Bioseguridad) and the local government authorization (approval with reference number PI-022/15).

### Availability of data.

All raw reads and assemblies were deposited in the NCBI database under BioProject number PRJ282520; BioSample accession numbers for each strain are included in Data Set S1B and C.
