# Supplementary figures and images for: Antagonistic Pleiotropy in the Bifunctional Surface Protein FadL (OmpP1) during Adaptation of Haemophilus influenzae to Chronic Lung Infection Associated with Chronic Obstructive Pulmonary Disease
Source: mBio. 2018 Sep 25;9(5):e01176-18. doi: 10.1128/mBio.01176-18 (PMC6156194; doi:10.1128/mBio.01176-18)

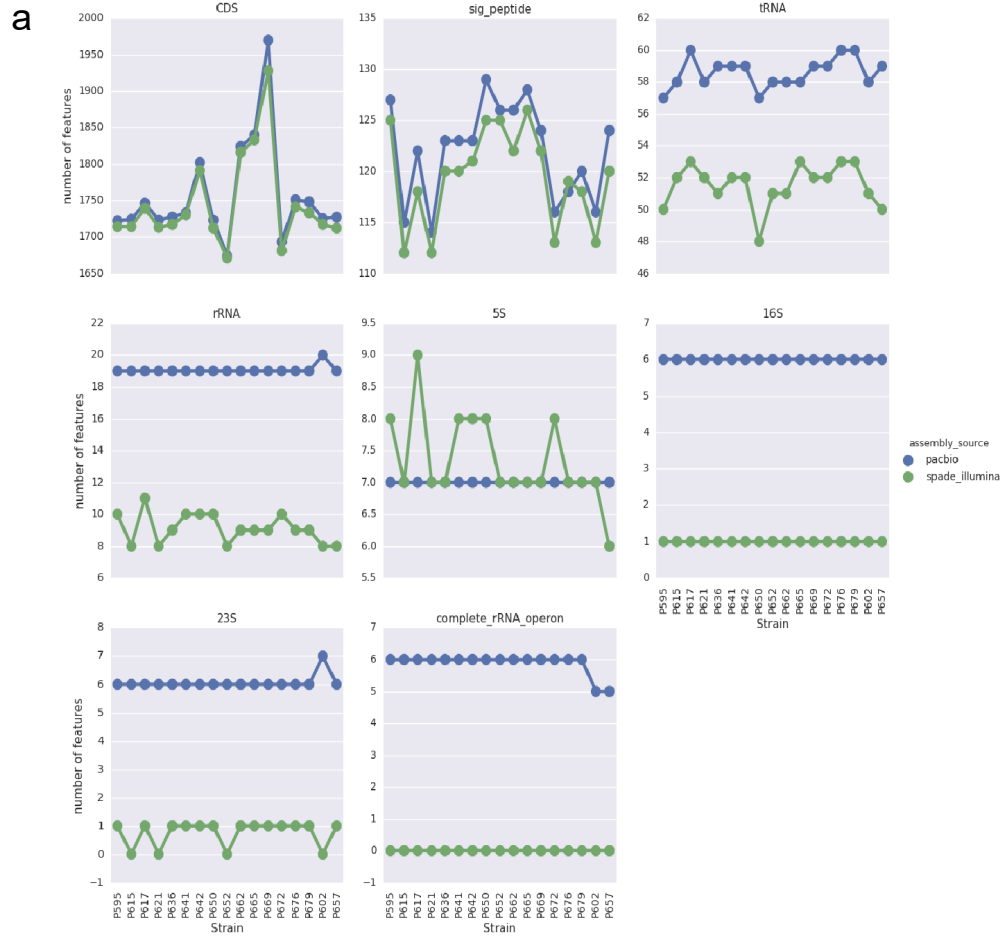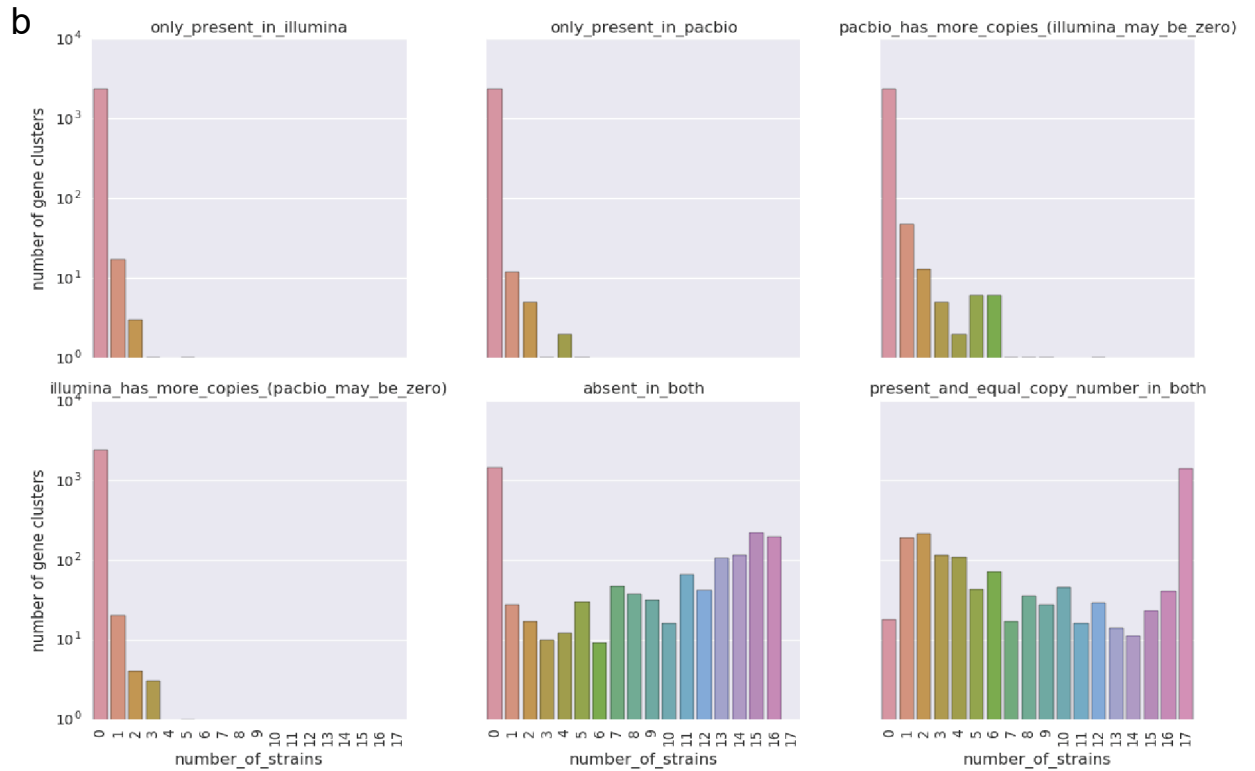

Supplement: FIG S1 [file mbo004184066sf1.pdf]

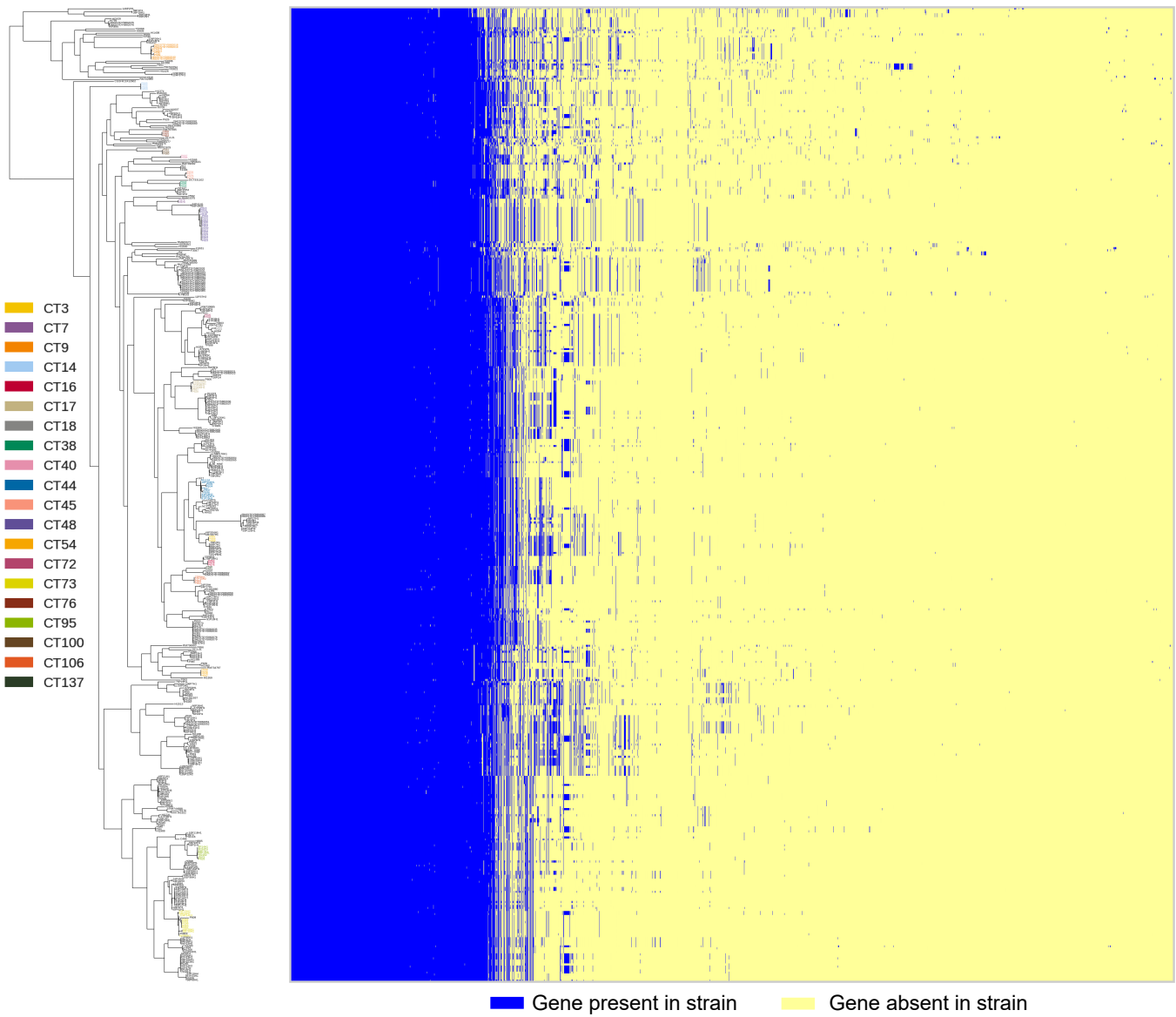

Supplement: FIG S2 [file mbo004184066sf2.pdf]

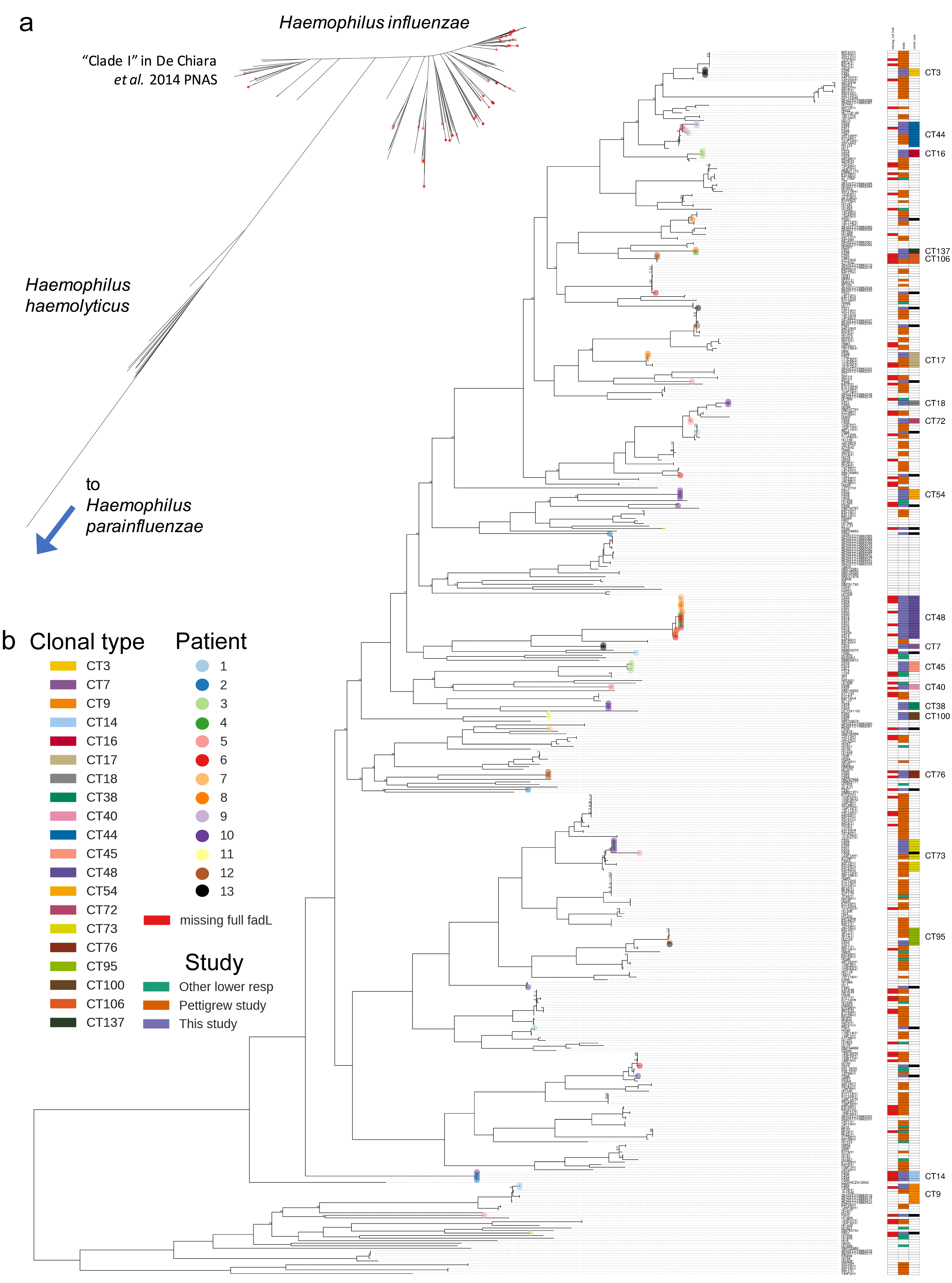

Supplement: FIG S4 [file mbo004184066sf4.gif]

a

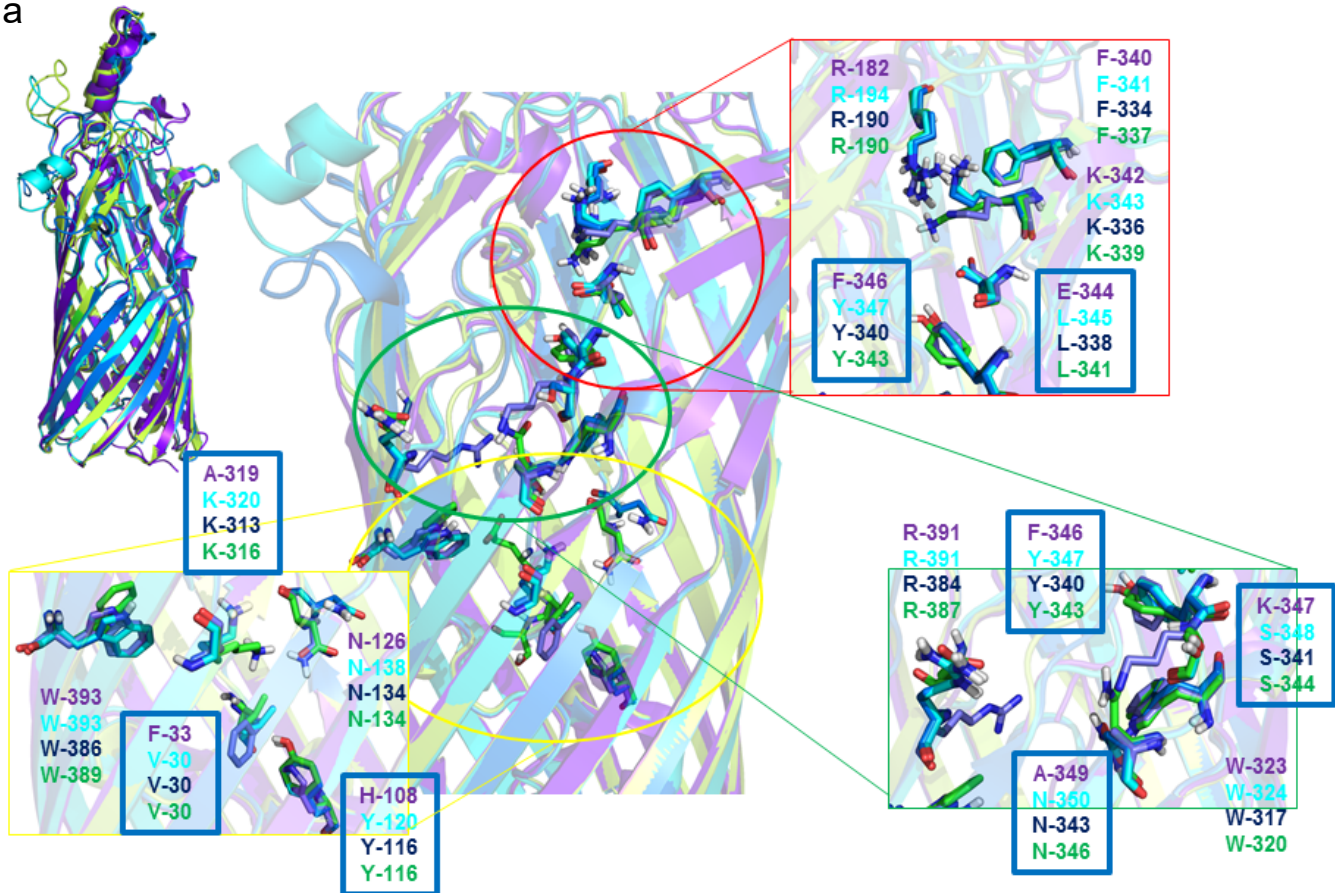

b

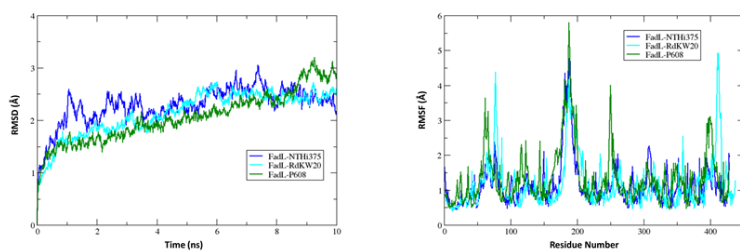

c

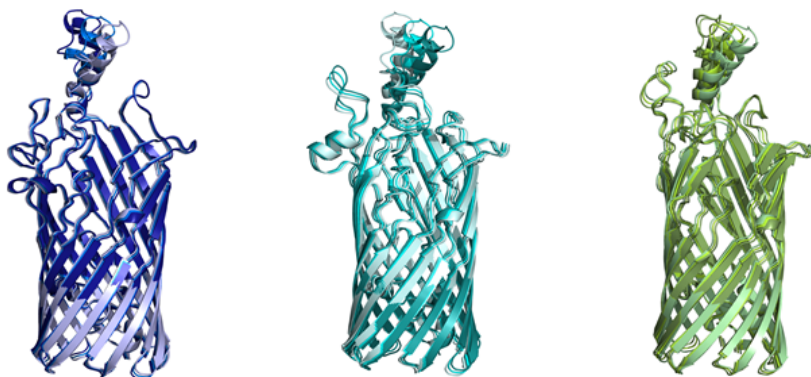

d

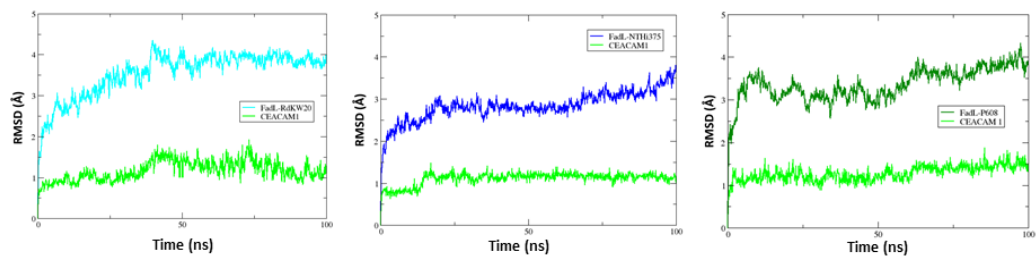

Supplement: FIG S5 [file mbo004184066sf5.pdf]

a

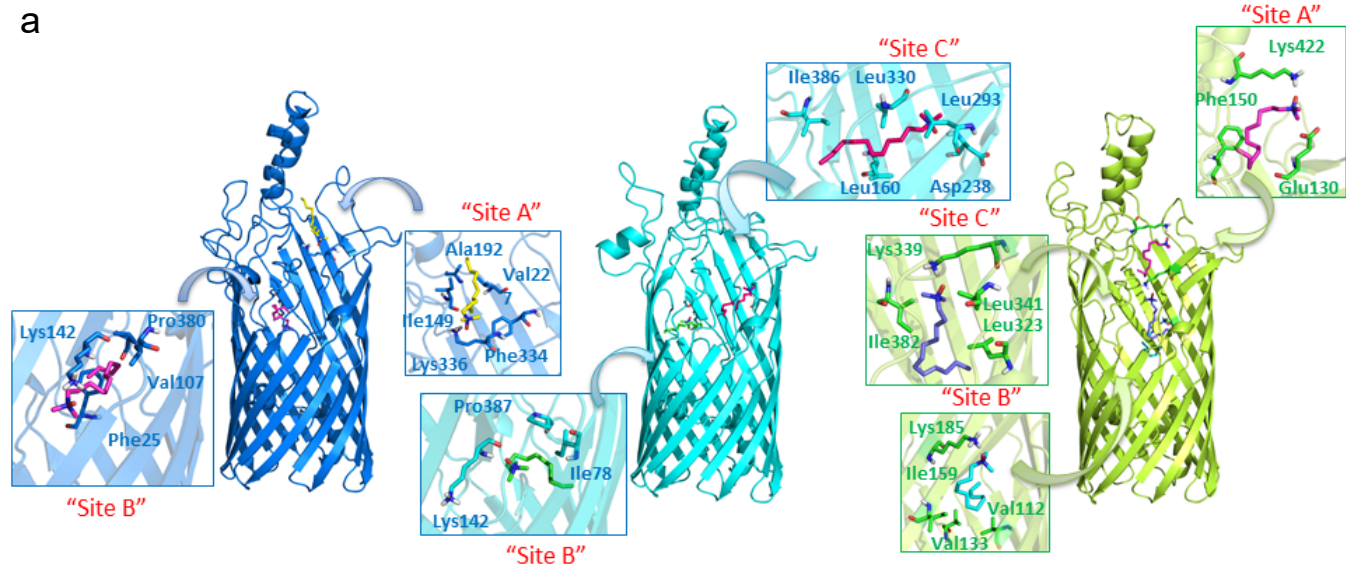

b

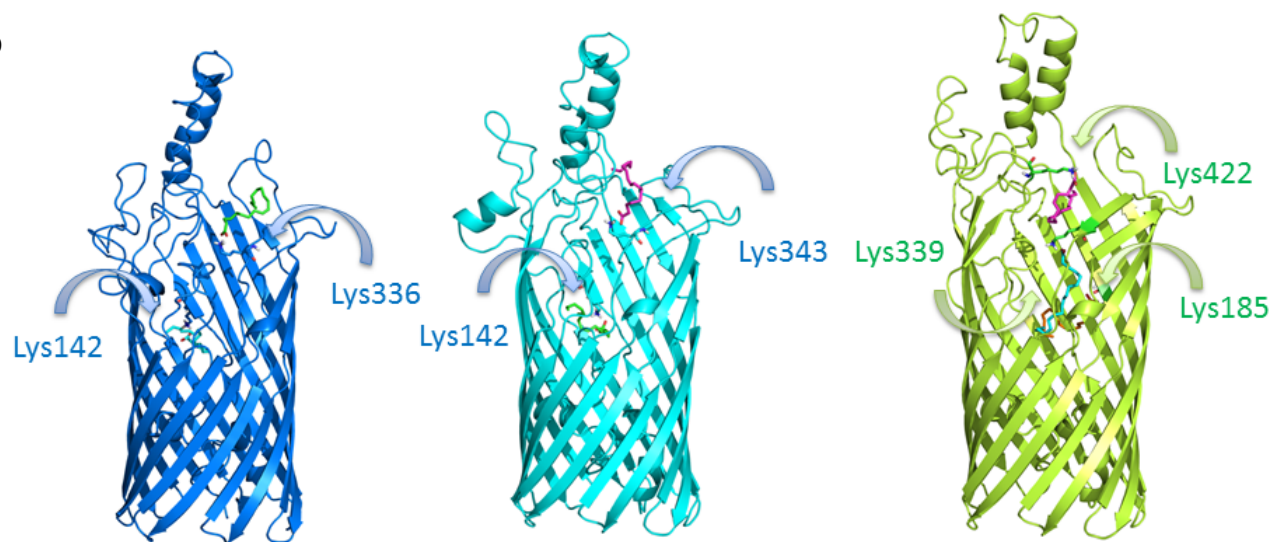

Supplement: FIG S6 [file mbo004184066sf6.pdf]
