# Supplementary material for: Antagonistic Pleiotropy in the Bifunctional Surface Protein FadL (OmpP1) during Adaptation of Haemophilus influenzae to Chronic Lung Infection Associated with Chronic Obstructive Pulmonary Disease
Source: mBio. 2018 Sep 25;9(5):e01176-18. doi: 10.1128/mBio.01176-18 (PMC6156194; doi:10.1128/mBio.01176-18)
Supplement: FIG S3 [file mbo004184066sf3.pdf]

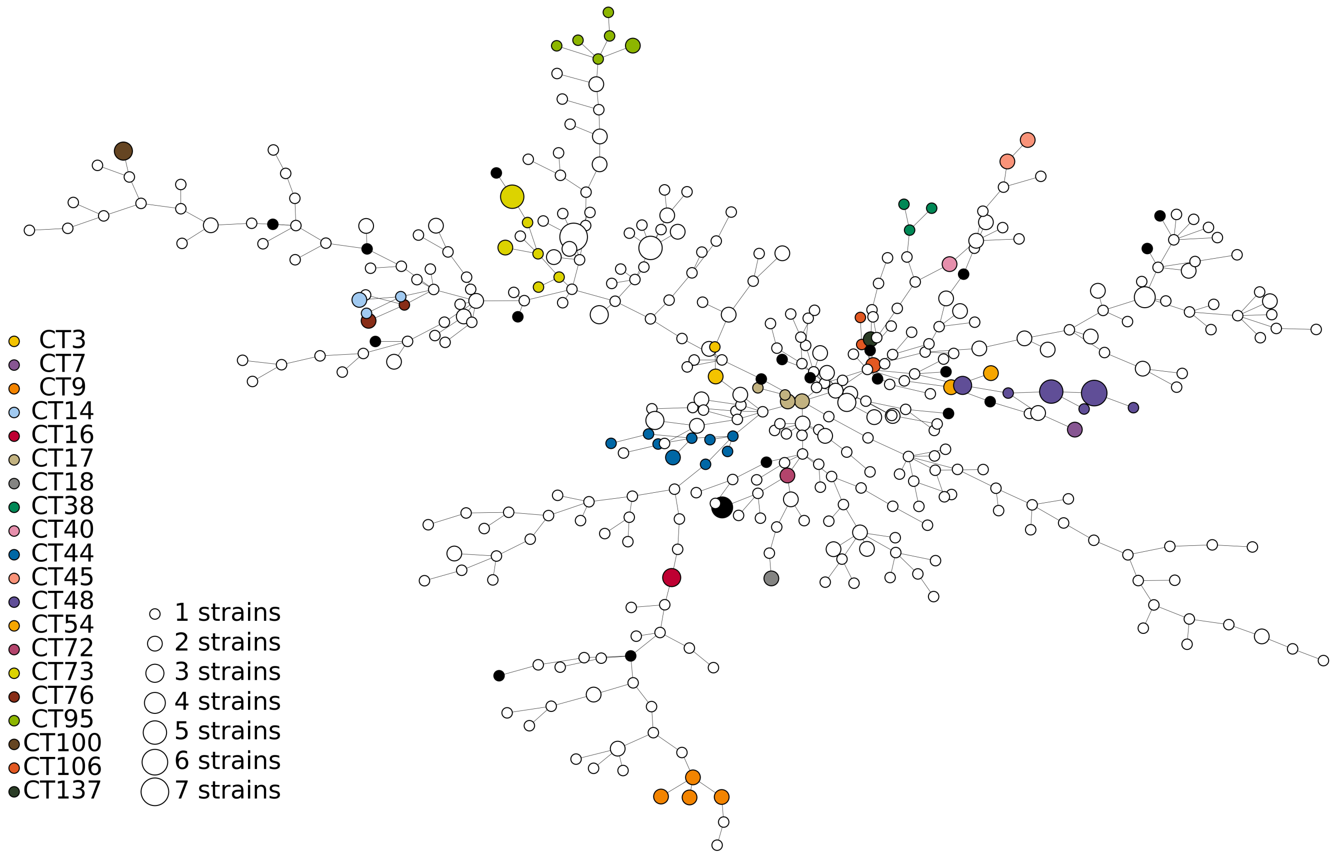
CT3
CT7
CT9
CT14
CT16
CT17
CT18
CT38
CT40
CT44
CT45
CT48
CT54
CT72
CT73
CT76
CT95
CT100
CT106
CT137
1 strains
2 strains
3 strains
4 strains
5 strains
6 strains
7 strains
