# Supplementary material for: Antagonistic Pleiotropy in the Bifunctional Surface Protein FadL (OmpP1) during Adaptation of Haemophilus influenzae to Chronic Lung Infection Associated with Chronic Obstructive Pulmonary Disease
Source: mBio. 2018 Sep 25;9(5):e01176-18. doi: 10.1128/mBio.01176-18 (PMC6156194; doi:10.1128/mBio.01176-18)
Supplement: TEXT S1 [file mbo004184066s1.docx]

**Supplementary Information Text for:**

**Antagonistic pleiotropy in the bifunctional surface protein FadL/P1 during adaptation of *Haemophilus influenzae* to chronic lung infection associated to COPD**

Javier Moleres, Ariadna Fernández-Calvet, Rachel L. Ehrlich, Sara Martí, Lucía Pérez-Regidor, Begoña Euba, Irene Rodríguez-Arce, Sergey Balashov, Ester Cuevas, Josefina Liñares, Carmen Ardanuy, Sonsoles Martín-Santamaría, Garth D. Ehrlich, Joshua C. Mell, Junkal Garmendia

#Address correspondence to:

Junkal Garmendia

Email: [juncal.garmendia@unavarra.es](mailto:juncal.garmendia@unavarra.es); [juncal.garmendia@csic.es](mailto:juncal.garmendia@csic.es)

Joshua Chang Mell

Email: [jcm385@drexel.edu](mailto:jcm385@drexel.edu)

**Supplementary Methods**

**DNA extractions.** A single colony from each clinical isolate was collected from chocolate agar and inoculated into 3 ml sBHI cultures. DNA was extracted after overnight growth with the DNeasy Blood and Tissue Kit (Qiagen, Cat. 69506) following manufacturer´s instructions. Purity, quality and concentration were measured by Nanodrop, agarose gel electrophoresis and Qbit fluorometry.

**Illlumina sequencing and assembly.** Nextera XT (Illumina) libraries were produced following manufacturer´s recommendations, and paired-end sequencing (2x151nt) was conducted on an Illumina NextSeq 500. Raw base call data (bcl) was converted into FastQ format (Illumina version 1.8) using the bcl2fastq conversion software (Illumina, version 2.17.1.14). *De novo* assembly of Illumina datasets used read pairs processed as follows: (i) reads were trimmed of adapters with Trimmomatic (v 0.33) (1); (ii) overlapping read pairs were merged with COPE (v 1.1.2) (2); (iii) reads were error corrected using ErrorCorrectReads.pl from allpathlg and (iv) finally, reads were assembled with SPAdes (v 3.7.0) (3).

**Pacific Biosciences sequencing and assembly.** SMRTbell libraries with mean insert size of ~6 kb were prepared according to manufacturer’s specifications. PacBio sequencing (v 2.1.0) was performed with one or two genomes per SMRTcell with P4-C2 chemistry. *De novo* assembly of PacBio data was done using SMRT pipe (v 2.3.0). SMRTcells with two genomes were demultiplexed based on barcodes. Initial assembly with the HGAP assembler (4) yielded single contigs with mean coverage from 54 to 66-fold. Genomes were circularized and permuted to the *dnaA* gene using Circlator (1.0.2), followed by Quiver-based error correction (4) and identification of DNA modification motifs using RS Modification and Motif Analysis.

**Reference assemblies from public databases.** *H. influenzae* assemblies available at NCBI were downloaded on June 23, 2016, and these were reconciled with a partially overlapping set of genomes available at the Sanger Institute (5), keeping the assembly with the fewest contigs. An additional 269 genomes from Pettigrew *et al.* (36) were added when they became available, and 30 genomes of *H. influenzae* sister species *H. haemolyticus* and *H. parainfluenzae* were included as outgroups. Assemblies were taxonomically classified using Taxator-tk, which found that several genomes from NCBI were more accurately classified as *H. haemolyticus* (strains156_HINF, 159_HINF, 781_HINF, 839_HINF, 167_HINF)*.* Strain provenance was encoded as “lower respiratory” if isolated from a sputum sample and annotated as from either COPD or pneumonia. Most analyses only excluded assemblies with >50 contigs (or >100 contigs for the Pettigrew *et al.* set).

**Homologous gene clustering.** Roary v3.5.1 was used to cluster homologous protein-coding genes from across the full set of assemblies and annotations (**Materials and** **Methods**). After testing blastp thresholds from 50% to 95% in increments of 5%, 75% amino acid identity was chosen for clustering because it was the highest threshold before the rapid increase in the number of rare genes. Contig breaks and chromosomal rearrangements cause Roary to sometimes over-split clusters into separate paralogous clusters based on synteny in adjacent genes, so following (6), we generated both “split” and “merged” gene possession tables; analyses presented all used “merged” gene possession tables, in which paralog splitting was reversed to generate homologous sets. Included in this set were genomes from outgroup sister species *H. haemolyticus* and *H. parainfluenzae* to provide outgroups. Thus, for clonal typing using goeBurst, a set of 309 protein-coding gene clusters were used, since these were present once and only once in all assemblies.

**Clonal typing.** For each single copy core gene, unique nucleotide sequences were defined as distinct alleles, and strings of allele IDs for each isolate were used as MLST-like input. The goeBurst full MST algorithm was used to produce an MST where the edge weights were distances between strains. A clustering threshold was chosen such that phylogenetically related strains clustered together, so edges with weights >15 were dropped from the graph and each resulting connected component was assigned an arbitrary clonal type (CT) number.

**Knockouts of *fadL***. To disrupt the *fadL* gene, a DNA fragment containing the *fadL* gene and its respective flanks (2,222 bp in total) was PCR amplified with *Phusion* DNA Polymerase (Thermofisher) using genomic DNA from NTHi375 as a template and primers fadL-F1/1302 and fadL-R1/1303 (**Table 4**). The gene containing PCR product was cloned into pJET1.2/blunt (Thermofisher), generating pJET1.2-*fadL*. A Spec^r^ cassette was PCR amplified from pRSM2832 using gene-specific mutagenic primers fadL-F2/1358 and fadL-R2/1359 (7). Primers were designed to delete sequences between the start codon and the last 7 codons of *fadL*. *E. coli* SW102 cells were prepared for recombineering, co-electroporated with pJET1.2-*fadL* (Amp^r^) (50 ng) and the *fadL*-specific mutagenic cassette (Spec^r^) (200 ng), as previously described (8). Mutagenized clones containing pJET1.2-*fadL::spec* were selected on LB agar with Amp_100_, Spec_50_. This plasmid was used as template to amplify the *fadL::spec* disruption cassette with primers fadL-F1/1302 and fadL-R1/1303, which was used to transform NTHi375 and RdKW20 using the MIV method. Transformants were selected on sBHI agar with Spec_30_, to obtain NTHi375Δ*fadL* and RdKW20Δ*fadL* mutant strains. Disruption was confirmed by PCR.

**SDS-PAGE protein separation and identification.** Whole cell extracts were prepared from bacterial suspensions recovered from chocolate agar plates, adjusted to OD_600_=1 in PBS, lysed by ultrasounds sonication, two-fold diluted with 2X loading buffer (Tris-HCl 62.5mM pH 6.8, SDS 2% w/v, glycerol 10%, DTT 50 mM; Bromophenol Blue 0.01% w/v), and heated to 95ºC for 5 min. Electrophoresis was performed at 15 mA until samples reached resolution phase (~2,5 h) and then at 30 mA for 16 h. FadL was identified in strain P589 by peptide mass fingerprinting (MALDI-TOF/TOF) at the Centro Nacional de Biotecnología (CNB-CSIC, Spain) proteomics facility.

**Cell culture and bacterial invasion assays.** For infection, PBS-normalized bacterial suspensions (OD_600_=1) were prepared by using NTHi strains grown on chocolate agar. A multiplicity of infection (MOI) of ~100:1 was used. To monitor invasion, cells were incubated with bacteria for 2 h in 1 ml EBSS (Earle's Balanced Salt Solution, Gibco), washed 3 times with PBS, and incubated for 1 h with RPMI 1640 medium containing 10% FCS, Hepes 10 mM and gentamicin 200 µg/ml to kill extracellular bacteria. Cells were washed 3 times with PBS, lysed with 300 µl PBS-saponin 0.025% for 10 min at room temperature, and serial dilutions plated on sBHI agar. Infections were performed in triplicate at least three independent times (n>9). Results are expressed as c.f.u./well.

**RNA extraction and real-time quantitative PCR (RT-qPCR) analysis.** NTHi strains were grown on chocolate agar. Bacteria (2 to 5 colonies) were inoculated into 20 ml sBHI, grown for 11 h, diluted into 40 ml fresh sBHI to OD_600_=0.05, and grown to OD_600_=0.6. Bacterial total RNA was isolated using TRIzol reagent (Invitrogen). Total RNA quality was evaluated using RNA 6000 Nano LabChips (Agilent 2100 Bioanalyzer). All samples had intact 16S and 23S ribosomal RNA. Complementary DNA (cDNA) was synthesized from total RNA (1 µg) using PrimerScript RT Reagent kit (Takara). Real-time quantitative PCR was performed using 1X SYBR Premix Ex Taq II (Tli RNaseH Plus, Takara) and primer mix; fluorescence data were analyzed with AriaMx Real-Time PCR System (Agilent Technologies). Relative quantities of mRNAs were calculated using the comparative threshold cycle (Ct) method and normalized using the *gyrA* gene as an endogenous control for each strain. Specific primers were designed with Primer Express software: for strain P589, fadL-qPCR-F3/1659 and fadL-qPCR-R1/1607; for P600, fadL-qPCR-F1/1605 and fadL-qPCR-R2/1608; for P608, fadL-qPCR-F2/1606 and fadL-qPCR-R1/1607; for strains NTHi375, Hi RdKW20, P634 and P666, fadL-qPCR-F1/1605 and fadL-qPCR-R1/1607 (see **Table 4**). Measures were performed in duplicate and at least three times (n≥6).

**Bacterial growth in chemically defined minimal medium.** Bacterial suspensions recovered with 1 ml MM-FFA (chemically defined minimal medium free of fatty acids) from freshly grown chocolate agar plates were adjusted to OD_600_=1. MM-FFA consists of MIV solutions S21, S22, S23 and S24 (100:1:1:1) (9), 10 µg/ml hemin and 10 µg/ml NAD. Arachidonic acid (AA) stock solution (100 mM) was prepared and diluted to the required working concentrations in ethanol. Then, 160 μl of (i) MM-FFA, (ii) MM-FFA with AA 25 μM, (iii) MM-FFA with fatty acid vehicle solution, i.e. ethanol volumes identical to those used for AA 25 μM, (iv) MM-FFA with glucose 20 mM, were transferred to individual wells in 96-well microtiter plates (Sarstedt). Next, 40 μl of the previously prepared bacterial suspension were added to each well. Plates were incubated in a SpectraMAX 340 microplate reader at 37 ºC, and OD_600_ was recorded every 30 min for 12 h. Experiments were performed in quadruplicate on three independent occasions (n=12).

**Fatty acid susceptibility testing.** NTHi strains grown on chocolate agar for 16 h were resuspended to OD_600_=0.1 in MM-FFA with 10 μg/ml hemin and 10 μg/ml NAD. As needed, MM-FFA was supplemented with 20 mM glucose, vehicle solution (96% ethanol), or free fatty acids (dissolved in ethanol to 100 mM stocks) at varying concentrations (oleic acid (OA), from 0.8 to 2 mM; AA, from 12.5 to 75 µM) (Sigma-Aldrich). OA and AA stock solutions (100 mM) were prepared and diluted to the required working concentrations in ethanol. MM-FFA with fatty acid (160 μl) were transferred to individual wells in 96-well microtiter plates (Sarstedt); 40 μl of the previously prepared bacterial suspensions were added to each well, and incubated for 20 h at 37ºC with 5% CO_2_ in static conditions. Vehicle solution, consisting of an ethanol volume equivalent to that used for the highest fatty acid concentration tested, and controls with MM-FFA only were performed in parallel. After incubation, bacteria were serially ten-fold diluted in PBS and plated on sBHI agar. Results are expressed as the mean+SEM of the percentage of bacterial survival ([c.f.u.ml^-1^_LCFA_/c.f.u.ml^-1^_vehicle solution_] x 100). Experiments were performed in triplicate on at least four independent occasions (n>12).

**NTHi mouse lung infection.** CD1 female mice (18-20 g) aged 4 to 5 weeks were purchased from Charles River Laboratories (France), housed under pathogen-free conditions at the Institute of Agrobiotechnology facilities (registration number ES/31-2016-000002-CR-SU-US), and used at 22-25 g. Animal handling and procedures were in accordance with the current European (Directive 86/609/EEC) and National (Real Decreto 53/2013) legislations, following the FELASA and ARRIVE guidelines, and with the approval of the Universidad Pública de Navarra (UPNa) Animal Experimentation Committee (Comité de Ética, Experimentación Animal y Bioseguridad) and the local Government authorization. NTHi375 and RdKW20, WT and Δ*fadL* strains, were used for lung infection. Infecting bacteria were previously grown on chocolate agar. For intranasal infection, 20 μl of a NTHi suspension containing ~2×10^8^ c.f.u. were placed at the entrance of the nostrils of each mouse until complete inhalation, in mice previously anesthetized with ketamine-xylacine (3:1). At 24 and 48 hpi, mice were euthanized using cervical dislocation. Lungs were homogenized with PBS (1:10 w/v), serially ten-fold diluted in PBS, and plated on sBHi agar for viable bacterial counts. Bronchoalveolar lavage fluid (BALF) samples were obtained by perfusion and collection of 0.7 ml of PBS, with help of a sterile 20G (1.1 mm diameter) Vialon^TM^ intravenous catheter (Becton-Dickinson) inserted into the trachea. An aliquot of each recovered BALF sample was serially ten-fold diluted in PBS and plated on sBHI agar to determine the number of viable bacteria. Results are expressed as mean ± SD of individual log_10_ c.f.u./sample (≤10 samples per group).

**Statistical analysis.** For cell infection and bacterial loads in lungs and BALF samples, statistical comparison of means were performed using the two-tail *t* test. A two-way ANOVA was used for statistical comparison, within rows, of column mean (Tukey’s multiple comparisons test) for fatty acid bactericidal assays. In all cases, p<0.05 value was considered statistically significant. Analyses were performed using Prism software, version 7 for Mac (GraphPad Software) statistical package.

**Molecular modeling methods**

***Homology modeling.*** Three FadL_NTHi_ variants, FadL_NTHi375_, FadL_RdKW20_ and FadL_P608_, were considered for computational 3D structures calculation by homology modeling. FadL coding sequences of strains RdKW20 (access. No. NC_000907) and NTHi375 (access. No. NZ_CP009610.1) were extracted from NCBI (http://www.ncbi.nlm.nih.gov). For the building of these models, FadL from *E. coli* (PDB-ID: 1T16) was used as a template. For each sequence, four homology models were generated using four different servers: SWISS-MODEL (10-13), I-TASSER (14-16), PHYRE2 (17), and RAPTOR X (18). The models derived from I-TASSER, PHYRE-2, and RAPTOR X were discarded because the structures did not have coherence with other experimental 3D structures from homologous proteins. Thus, computational studies were performed using the 3D structures obtained from SWISS-MODEL, which were submitted to molecular dynamics (MD) simulations to optimize the geometry and study the stability.

***Molecular dynamics simulations.*** We assessed the stability of the homology models, the mobility of the loops and the stability of the binding poses, by means of MD simulations as implemented in AMBER 14 suite of programs (19). Missing hydrogen atoms were added, and protonation state of ionisable groups was computed by using Maestro Protein Preparation Wizard (20-23). Atom types and charges were assigned according to AMBER ff10 force field. The homology models, together with the structure from PDB 1IT16, were hydrated by using cubic boxes containing explicit TIP3P water molecules extending 10 Å away from any protein atom for simulating the aqueous environment with the help of AmberTools (19) with added counter ions to neutralize the system. Minimization was performed using Sander and MD simulations were run using the pmemd, which are distributed within the AMBER 14 package. The smooth particle mesh Ewald method was used to represent the electrostatic attractions in the system while each simulation was under periodic boundary conditions, and the grid spacing was 1 Å. Initial annealing of the system occurred steadily and lightly from 100 °K to 300 °K over 25 ps. Temperature was kept constant at 300 °K during 50 ps with progressive energy minimizations and also a solute restraint. The restraints were gradually released by the solute, which was closely followed by a 20 ps heating period which went from 100 °K to 300 °K, once completed the restraints were removed. Each of the simulations lasted 10 ns. The systems then advanced in an isothermal-isobaric ensemble. Long-range electrostatic interactions were accounted for by means of the particle mesh Ewald approach applying periodic boundary conditions. The root mean square deviation (RMSD) as a function of time with respect to the starting structure for the Cα atoms was computed using CPPTRAJ.

***Normal mode analysis.*** To compute the low frequency normal modes of FadL_NTHi_ models, the elastic network model was used with the Web interface ElNemo (24). The starting geometries were those from the MD simulations. From the resulting normal mode (NM) conformations, we selected three structures for docking purposes: the one corresponding to the crystallographic structure (NM-6), the one corresponding to the last structure of the displacement in one of the directions of the normal mode (NM-1), and the last located structure in the opposite direction (NM-11). The three structures were submitted to 100 000 steps of steepest descent minimization with MacroModel and optimized with AMBER force field, before being used for docking calculations.

***Ligand-protein docking calculations***

*Preparation of the macromolecules.* 3D coordinates of hCEACAM1 and the FadL homology models were used for docking purposes. Quality of the minimized models was verified using the PDBSUM online resource, which displays Ramachandran plot of the structures. Models were prepared for docking calculations by adding Kollman charges with the help of AutoDockTools (19).

*Building of ligands.* The 3D structure of lauryl dimethylamine-*N*-oxide (LDA) was extracted from the crystallographic structure PDB-ID 1T16. The 3D coordinates of AA and OA were built with Maestro (20-23). The geometries of the three ligands were optimized with MMFFs force field by using MacroModel. Ligands were prepared for docking calculations using AutoDockTools, setting all rotatable bonds free to move during the docking calculation.

*Docking calculations*. Docking calculations of all compounds were performed by using AutoDock 4.2.2. (25). Analysis was performed with AutoDockTools. The grid point spacing was set at 0.375 Ǻ, and a hexahedral box was built with x, y, z dimensions FadL: 122 Ǻ, 108 Ǻ, 92 Ǻ centered in the centroid position among residues Arg161, Lys336 and Ala378 for FadL_NTHi375_; Arg194, Lys343 and Ala385 for FadL_RdKW20_; Arg190, Lys339 and Ala381 for FadL_P608_. A total of 200 runs using Lamarckian Genetic algorithm was performed, with a population size of 100, and 250000 energy evaluations.

**Supplementary Results—Computational Modeling**

***Homology modeling*.** FadL_NTHi375_ FadL_RdKW20_ and FadL_P608_ were considered for computational 3D structures calculation by homology modeling. FadL coding sequences of strains RdKW20 and NTHi375 were extracted from NCBI. FadL from *E. coli* (PDB-ID: 1T16) was used as a template (26). For each sequence, four homology models were generated using four different servers: SWISS-MODEL, I-TASSER, PHYRE2 and RAPTOR X. The models derived from I-TASSER, PHYRE-2, and RAPTOR X were discarded because the structures did not have coherence with other experimental 3D structures from homologous proteins. The 3D structures of the computed structures were very similar between them, and also in comparison to *E. coli* FadL. Detailed analysis was performed, focusing on the inner part of the β-barrel and the flaking loops. Regarding the interior of the β-barrel, some different sequence amino acids in comparison to *E. coli* FadL were identified, pointing to a putative role in the recognition of fatty acids (**Fig. S5A**).

Thus, computational studies were performed using the 3D structures obtained from SWISS-MODEL, which were submitted to molecular dynamics (MD) simulations to optimize the geometry. The three modeled structures showed high stability (**Fig. S5B**). Normal mode analysis (NMA) was also performed to study the flexibility of the loops and the three models exhibited high coherence among them thus confirming the stability (**Fig. S5C**).

***Docking of FadL and hCEACAM1.*** We performed protein-protein docking with ZDOCK Server (27), between each of the FadL_NTHi375,_ FadL_RdKW20_, and FadL_P608_ models with hCEACAM1 (PDB-ID: 4QXW). The resulting protein-protein complexes were submitted to 100ns MD simulations being possible to observe high stability for the three complexes along the simulation (**Fig. S5D**).

Protein-protein interaction analysis of the FadL_NTHi375_/hCEACAM1 complex identified relevant polar interactions between Lys213(FadL_NTHi375_)-Glu99(hCEACAM1), Ala334-Gln27, Ser276-Gly51, and also hydrophobic interactions between Ile216-Ile91. These interactions remained stable along the MD simulation. In the case of Lys213(FadL_NTHi375_), new interactions were established with residues Gln89 and Asn97 from hCEACAM1, strengthening the protein-protein complex. Also remained stable along the MD simulation the CH-π interaction between Tyr34-Val39 both from hCEACAM1, hydrogen bond between Gln44 and OH from Tyr34, Gln44 with O from Ser32, Gln89-Ser32 and Gln89-Tyr34. The MD simulation of the FadL_RdKW20_/hCEACAM1 complex also led to a stable protein-protein complex where the main interactions were polar contacts between Lys225(FadL_RdKW20_)-Asp40(hCEACAM1), Phe222-Gly41, Lys290-Ser93 and Lys339-Asp94, and hydrophobic interactions between Ile286-Ile91 side chains. Also remained stable along the MD simulation the CH-π interaction between Tyr34-Val39 both from hCEACAM1, hydrogen bond between Gln44 and OH from Tyr34, Gln44 with O from Ser32, and Gln89-Tyr34. Protein-protein interaction analysis of the FadL_P608_/hCEACAM1 complex identified relevant polar interactions between Lys216(FadL_P608_)-Gln89(hCEACAM1), Lys217-Glu99, Asn200-Gln1, Gly193-Asp94, Asp332-Tyr48 and Gly279-Gly51; also CH-π interaction between Trp220-Ile91 and hydrophobic interactions between Val196-Val96 and Leu214-Val39. These interactions remained stable along the MD simulation. Also remained stable along the MD simulation the CH-π interaction between Tyr34-Val39 both from hCEACAM1, hydrogen bond between Gln44 and OH from Tyr34, Gln44 with O from Ser32, and Gln89-Tyr34. Interestingly, the residues from hCEACAM1 (residues Ser32, Tyr34, Val39, Gln44, Gln89 and Ile91) that interact with FadL were previously reported to be crucial for the interaction with pathogen components (28). Our computational studies clearly identify these particular residues as directly involved in the interaction with FadL, suggesting they shape a region relevant for the molecular recognition (see **Fig. 6**).

***Docking calculations of AA, LDA and OA***. Docking calculations of AA, OA and LDA were performed in the three modeled structures, FAdL_NTHI375_ and FadL_RdKW20_, and FadL_P608_. Results of the docking studies of AA inside FAdL_NTHI375_ and FadL_RdKW20_ are reported in the main text (**Fig. 7**). We here detail the description of the different predicted binding poses and the interactions. Calculations predicted two AA binding modes with good theoretical binding energies for the FadL_NTHi375_ model. One binding mode places AA at the entrance of the pocket, in a hydrophobic groove located between L3 and L4 (site A), where AA is bound and solvent accessible. Interactions with polar residues, specifically with Lys336 (L5), lipophilic interactions with Ile278, and CH-π interactions with Phe150 and Phe334 side chains were identified. A second binding mode for AA was predicted in the FadL_NTHi375_ model at the deep of the β-barrel, establishing polar contact with Lys142 present in L2 (site B), which has a kink that points inward. Interestingly, these binding poses were also found for FadL_RdKW20_ model. Docking of AA was also performed inside the FadL_P608_ structure. In this case, apart from the docked poses in the binding sites A and B, an additional binding pose was found in a third binding site (site C) within a hydrophobic pocket of the β-barrel, where the carboxylate group establishes a hydrogen bond with the Lys339, and hydrophobic interactions are established with Phe135, Leu341 and Ile382 side chains (**Fig. 7**).

We also performed docking calculations with LDA in three modeled structures FadL_NTHi375,_ FadL_RdKW20_, and FadL_P608_. For the FadL_NTHi375_ model, two LDA binding modes were predicted with good theoretical binding energies. One binding mode places LDA at the entrance of the pocket, in a hydrophobic groove located between L3 and L4 (site A), where LDA is bound and solvent accessible. Interactions with polar residues, specifically with Lys336 (L5), lipophilic interactions with the hydrophobic pocket delimited by Ile149, Ile195, Val227, Leu273 and Ile278, and CH-π interactions with Phe150 were identified (**Fig. S6A**). A second binding mode for LDA was predicted in the FadL_NTHi375_ model at the deep of the β-barrel, establishing polar contact with Lys138 present in L2 (site B), which has a kink that points inward (**Fig. S6A**). This binding pose was also found for LDA in the FadL_RdKW20_ model. In that case, an additional binding pose was found in a third binding site (site C) within a hydrophobic pocket of the β-barrel, where the N-oxide group of LDA establishes a hydrogen bond polar interaction with the Asp238 NH group, and hydrophobic interactions are established with Phe25, Leu160, Val192, Leu236 and Leu295 side chains (**Fig. S6A**). These three binding poses (sites A, B and C) were found also for FadL_P608_. Our docked poses for LDA in the FadL models and the X-ray crystallographic pose structure (PDB-ID: 1T16) present some differences. LDA binds in two sites in the FadL*_E. coli_* crystal complex, at the hydrophobic pocket delimited by the positively charged residues Arg182 and Lys342, and another site located close to Arg391, where LDA establishes polar interactions. None of the docked solutions obtained for the FadL models were predicted to bind to these sites.

We were also prompted to perform docking calculations of OA in the 3D models of FadL_NTHi375_ and FadL_RdKW20_, leading to predicted binding poses at sites A and B (**Fig. S6B**). At site C, we did not find any docked pose. The main interactions involved polar residues between L3 and L4 (site A), such as Lys336 (for FadL_NTHi375_) and Lys343 (for FadL_RdKW20_), and polar interactions with Lys138 (for FadL_NTHi375_) and Lys142 (for FadL_RdKW20_) and present in L2 (site B). Overall, the docked poses for OA, at both sites A and B, were very similar to those obtained for LDA. However, docking calculations of OA in the 3D model FadL_P608_ led to predicted binding poses also at site C within a hydrophobic pocket of the β-barrel involved main polar interactions with Lys339 (for FadL_P608_).

**References**

1. Bolger AM, Lohse M, Usadel B. 2014. Trimmomatic: a flexible trimmer for Illumina sequence data. Bioinformatics 30:2114-20.

2. Liu B, Yuan J, Yiu SM, Li Z, Xie Y, Chen Y, Shi Y, Zhang H, Li Y, Lam TW, Luo R. 2012. COPE: an accurate k-mer-based pair-end reads connection tool to facilitate genome assembly. Bioinformatics 28:2870-4.

3. Bankevich A, Nurk S, Antipov D, Gurevich AA, Dvorkin M, Kulikov AS, Lesin VM, Nikolenko SI, Pham S, Prjibelski AD, Pyshkin AV, Sirotkin AV, Vyahhi N, Tesler G, Alekseyev MA, Pevzner PA. 2012. SPAdes: a new genome assembly algorithm and its applications to single-cell sequencing. J Comput Biol 19:455-77.

4. Chin CS, Alexander DH, Marks P, Klammer AA, Drake J, Heiner C, Clum A, Copeland A, Huddleston J, Eichler EE, Turner SW, Korlach J. 2013. Nonhybrid, finished microbial genome assemblies from long-read SMRT sequencing data. Nat Methods 10:563-9.

5. De Chiara M, Hood D, Muzzi A, Pickard DJ, Perkins T, Pizza M, Dougan G, Rappuoli R, Moxon ER, Soriani M, Donati C. 2014. Genome sequencing of disease and carriage isolates of nontypeable *Haemophilus influenzae* identifies discrete population structure. Proc Natl Acad Sci U S A 111:5439-44.

6. Lee AH, Flibotte S, Sinha S, Paiero A, Ehrlich RL, Balashov S, Ehrlich GD, Zlosnik JE, Mell JC, Nislow C. 2017. Phenotypic diversity and genotypic flexibility of *Burkholderia cenocepacia* during long-term chronic infection of cystic fibrosis lungs. Genome Res 27:650-662.

7. Tracy E, Ye F, Baker BD, Munson RS, Jr. 2008. Construction of non-polar mutants in *Haemophilus influenzae* using FLP recombinase technology. BMC Mol Biol 9:101.

8. Sinha S, Mell JC, Redfield RJ. 2012. Seventeen Sxy-dependent cyclic AMP receptor protein site-regulated genes are needed for natural transformation in *Haemophilus influenzae*. J Bacteriol 194:5245-54.

9. Herriott RM, Meyer EM, Vogt M. 1970. Defined nongrowth media for stage II development of competence in Haemophilus influenzae. J Bacteriol 101:517-24.

10. Arnold K, Bordoli L, Kopp J, Schwede T. 2006. The SWISS-MODEL workspace: a web-based environment for protein structure homology modelling. Bioinformatics 22:195-201.

11. Biasini M, Bienert S, Waterhouse A, Arnold K, Studer G, Schmidt T, Kiefer F, Gallo Cassarino T, Bertoni M, Bordoli L, Schwede T. 2014. SWISS-MODEL: modelling protein tertiary and quaternary structure using evolutionary information. Nucleic Acids Res 42:W252-8.

12. Guex N, Peitsch MC, Schwede T. 2009. Automated comparative protein structure modeling with SWISS-MODEL and Swiss-PdbViewer: a historical perspective. Electrophoresis 30 Suppl 1:S162-73.

13. Kiefer F, Arnold K, Kunzli M, Bordoli L, Schwede T. 2009. The SWISS-MODEL Repository and associated resources. Nucleic Acids Res 37:D387-92.

14. Roy A, Kucukural A, Zhang Y. 2010. I-TASSER: a unified platform for automated protein structure and function prediction. Nat Protoc 5:725-38.

15. Yang J, Yan R, Roy A, Xu D, Poisson J, Zhang Y. 2015. The I-TASSER Suite: protein structure and function prediction. Nat Methods 12:7-8.

16. Zhang Y. 2008. I-TASSER server for protein 3D structure prediction. BMC Bioinformatics 9:40.

17. Kelley LA, Mezulis S, Yates CM, Wass MN, Sternberg MJ. 2015. The Phyre2 web portal for protein modeling, prediction and analysis. Nat Protoc 10:845-58.

18. Kallberg M, Wang H, Wang S, Peng J, Wang Z, Lu H, Xu J. 2012. Template-based protein structure modeling using the RaptorX web server. Nat Protoc 7:1511-22.

19. D.A. Case VB, J.T. Berryman, R.M. Betz, Q. Cai, D.S. Cerutti, T.E. Cheatham, III, T.A. Darden, R.E., Duke HG, A.W. Goetz, S. Gusarov, N. Homeyer, P. Janowski, J. Kaus, I. Kolossváry, A. Kovalenko,, T.S. Lee SL, T. Luchko, R. Luo, B. Madej, K.M. Merz, F. Paesani, D.R. Roe, A. Roitberg, C. Sagui,, R. Salomon-Ferrer GS, C.L. Simmerling, W. Smith, J. Swails, R.C. Walker, J. Wang, R.M. Wolf, X., Kollman. WaPA. 2014. AMBER 14, University of California, San Francisco.

20. Schrodinger. 2012. Epik version 2.3, Schrodinger, LLC, New York, NY.

21. Schrodinger. 2012. Impact version5.8, Schrodinger, LLC, New York, NY, 2012.

22. Schrodinger. 2012. Prime version 3.1, Schrodinger, LLC, New York, NY, 2012.

23. S S. 2012. Suite 2012: Schrodinger Suite 2012 Protein Preparation Wizard.

24. Suhre K, Sanejouand YH. 2004. ElNemo: a normal mode web server for protein movement analysis and the generation of templates for molecular replacement. Nucleic Acids Res 32:W610-4.

25. Morris GMG, D. S.; Halliday, R. S.; Huey, R.; Hart, W. E.; Belew, R. K.; Olson, A. J. 1998. AutoDock treats the ligand as a flexible unit and the protein as a rigid unit. J Comput Chem 19.

26. Notredame C, Higgins DG, Heringa J. 2000. T-Coffee: A novel method for fast and accurate multiple sequence alignment. J Mol Biol 302:205-17.

27. Pierce BG, Wiehe K, Hwang H, Kim BH, Vreven T, Weng Z. 2014. ZDOCK server: interactive docking prediction of protein-protein complexes and symmetric multimers. Bioinformatics 30:1771-3.

28. Villullas S, Hill DJ, Sessions RB, Rea J, Virji M. 2007. Mutational analysis of human CEACAM1: the potential of receptor polymorphism in increasing host susceptibility to bacterial infection. Cell Microbiol 9:329-46.
